# Solvent-Controlled Self-Assembled Oligopyrrolic Receptor †

**DOI:** 10.3390/molecules26061771

**Published:** 2021-03-22

**Authors:** Fei Wang, Kejiang Liang, Mads Christian Larsen, Steffen Bähring, Masatoshi Ishida, Hiroyuki Furuta, Atanu Jana

**Affiliations:** 1 Center for Supramolecular Chemistry and Catalysis, Department of Chemistry, Shanghai University, No. 333 Nanchen Road, Baoshan District, Shanghai 200444, China; wangfei532447234@163.com (F.W.); KejiangLiang@shu.edu.cn (K.L.); 2 Department of Physics, Chemistry and Pharmacy, University of Southern Denmark, Campusvej 55, 5230 Odense, Denmark; mclarsen@sdu.dk; 3 Center for Molecular Systems, Department of Chemistry and Biochemistry, Graduate School of Engineering, Kyushu University, Fukuoka 819-0395, Japan; 4 Department of Chemistry, Gandhi Institute of Technology and Management (GITAM), NH 207, Nagadenehalli, Doddaballapur Taluk, Bengaluru 561203, India

**Keywords:** supramolecular receptor, host–guest chemistry, colorimetric acid-sensing, oligopyrrole

## Abstract

We report a fully organic pyridine-tetrapyrrolic U-shaped acyclic receptor **10**, which prefers a supramolecular pseudo-macrocyclic dimeric structure (**10**)_2_ in a less polar, non-coordinating solvent (e.g., CHCl_3_). Conversely, when it is crystalized from a polar, coordinating solvent (e.g., *N*,*N*-dimethylformamide, DMF), it exhibited an infinite supramolecular one-dimensional (1D) “zig-zag” polymeric chain, as inferred from the single-crystal X-ray structures. This supramolecular system acts as a potential receptor for strong acids, e.g., *p*-toluenesulfonic acid (PTSA), methane sulfonic acid (MSA), H_2_SO_4_, HNO_3_, and HCl, with a prominent colorimetric response from pale yellow to deep red. The receptor can easily be recovered from the organic solution of the host–guest complex by simple aqueous washing. It was observed that relatively stronger acids with p*K*_a_ < −1.92 in water were able to interact with the receptor, as inferred from ^1^H NMR titration in tetrahydrofuran-*d_8_* (THF-*d*_8_) and ultraviolet–visible (UV–vis) spectroscopic titrations in anhydrous THF at 298 K. Therefore, this new dynamic supramolecular receptor system may have potentiality in materials science research.

## 1. Introduction

Supramolecular architectures, such as capsules, cages, and polymers, have proven their value by demonstrating functional properties, such as host–guest chemistry and catalysis [1,2,3,4,5], drug delivery [6,7,8,9], and anion binding [10,11,12], with high efficiencies and selectivity. Rebek and co-workers have pioneered this area by exploring dynamic hydrogen-bonded complementary receptor–guest systems [13,14,15]. After his epochal discovery of the first fully organic supramolecular “tennis-ball” and “molecular softball” used for various host–guest complexation studies and catalytic activities as “microreactors” for bimolecular reactions, several research groups have extensively studied discrete cage-like architectures employing metal–ligand self-assembly [16,17,18,19]. Meanwhile, pioneered by Fujita, several well-defined metallacages have been demonstrated as reaction containers for various catalytic reactions [20,21,22]. Similarly, supramolecular polymers have been demonstrated as anion and ion-pair receptors by Sessler and co-workers [23,24,25]. All of these fully organic supramolecular dynamic cages, robust metallacages, and polymeric materials are of great importance in the modern-day chemical research field for their unique chemical behaviors, which are used for (1) exclusively synthesizing a particular target product, (2) anion “capture and release,” (3) ion-pair recognition, (4) removal of specific pollutants, and (5) drug delivery for biomedical research. Therefore, the creation of new organic supramolecular receptors is in demand to understand the in-depth mechanism of various host–guest complexations and other potential applications of these self-assembled structures.

**Figure 1:**
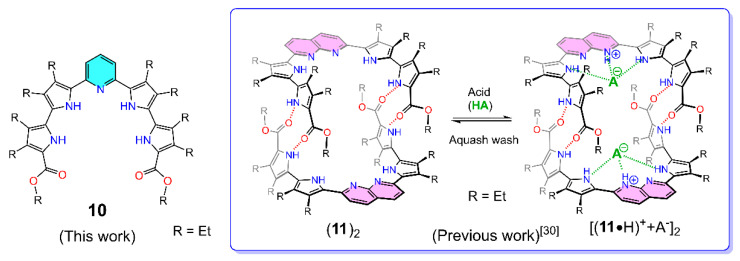
Chemical structures of the oligopyrrole-based receptor **10** used in our present study and receptor **11** used in our earlier study [30]. The pseudo-macrocyclic cage of a rigid dimer (**11**)_2_ formed via hydrogen-bonding interactions enables acid recognition within its cavity.

Strong organic acids (e.g., methane sulfonic acid (MSA), *p*-toluenesulfonic acid (PTSA), etc.) and mineral acids (e.g., HCl, HNO_3_, H_2_SO_4_, etc.) are common laboratory and industrial waste materials and are potential hazards. Therefore, capturing and sensing these acids with suitable receptors is an avenue that needs to be explored. Many such synthetic receptors are reported so far in the current literature, and can be used to sense and capture selective organic acids [26,27,28,29,30]. Recently, we have been interested in developing potential oligopyrrolic supramolecular receptors for acid recognition in an organic medium capable of releasing these acid guests upon simple aqueous wash. The strategy of making these kinds of acid receptors is principally based on the incorporation of pyridine- or naphthyridine-based heterocyclic cores into an oligopyrrolic molecular skeleton. Herein, we designed and synthesized such a pyridine-based tetrapyrrolic acyclic receptor unit (**10**), which self-assembles in the solid phase in two different forms depending on the solvent polarity and the ability to coordinate (Figure 1). Inspired by the successful results obtained from our earlier naphthyridine-based system (**11**) that was recently reported [30], we initially thought that the incorporation of pyridine (p*K*_a_ = 5.20 in water) into this new receptor skeleton used in the present study may provide a more basic environment than the naphthyridine- based (p*K*_a_ = 3.39 in water) receptor reported earlier. Similarly to the naphthyridine-based receptor **11**, the presence of sterically crowded ethyl groups at the *β*-positions of the pyrrole units in our present acyclic receptor system **10** imposes an orthogonal arrangement of terminal pyrrole units relative to the mean plane defined by the three central heterocyclic rings, as inferred from single-crystal X-ray structural analyses. This unique “building block” allows us to provide a suitable platform for constructing either a self-assembled dimeric cage (**10**)_2_ or an infinite polymer (**10**)_n_ via C=O···HN quadruple hydrogen-bonding interactions within neighboring acyclic units, as shown in the case of our earlier receptor **11** (see the inset of Figure 1 for details). Incorporating a terminal pyrrole that possesses an *α*-ester group provides an additional source of complementary hydrogen bond donors (i.e., NH moieties) and acceptors (i.e., C=O units) in the same molecular edifice. In addition to that, the NH protons of the adjacent pyrrole rings also help hydrogen-bonding interactions with the corresponding anions generated in the medium. It was speculated that such systems would allow us to provide a suitable skeleton for proton-coupled anion recognition studies. In pursuit of this goal, pyridine was positioned within the system, promoting the protonation step caused by suitable acids. Consequently, this new supramolecular pseudo-macrocyclic receptor provides further knowledge of strong acid sensing and recognition with high reversibility. 

## 2. Results and Discussion 

### 2.1. Synthesis

**Scheme 1:**
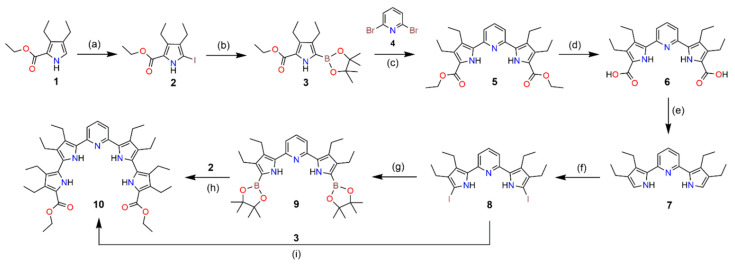
Synthetic scheme for acyclic receptor **10**. (**a**) I_2_/KI, EtOH/H_2_O, 85 °C, 8 h, Na_2_S_2_O_3_ (Yield: 88%). (**b**) HB(pin), Et_3_N, PdCl_2_(PPh_3_)_2_, 1,4-dioxane, 65 °C, 3 h (Yield: 92%). (**c**) Pd(OAc)_2_, PPh_3_, K_2_CO_3_, DMF/H_2_O, N_2_, reflux, 24 h (Yield: 83%). (**d**) NaOH, EtOH/H_2_O, 85 °C, 5 h (Yield: 95%). (**e**) Ethylene glycol, 185 °C, 50 min (Yield: 90%). (**f**) I_2_/KI, NaHCO_3_, CH_2_Cl_2_/H_2_O, rt, 1 h, Na_2_S_2_O_3_ (Yield: 85%). (**g**) HB(pin), Et_3_N, PdCl_2_(PPh_3_)_2_, 1,4-dioxane, 65 °C, 3 h (Yield: 89%). (**h**) Pd(PPh_3_)_4_, K_2_CO_3_, DMF/H_2_O, reflux, 16 h (Yield: 58%). (**i**) Pd(PPh_3_)_4_, K_2_CO_3_, DMF/H_2_O, reflux, 16 h (Yield: 55%).

The acyclic receptor **10** was synthesized via a multistep process, which is described below in Scheme 1. Precursor **3** was synthesized using procedures from the literature [30,31]. Compound **5** was synthesized via a Pd(II)-acetate-catalyzed Suzuki–Miyaura cross-coupling reaction using commercially available 2,6-dibromo-pyridine [32]. Hydrolysis of compound **5** with NaOH solution was followed by the decarboxylation of compound **6** at both the terminal pyrroles to produce a highly reactive intermediate compound **7**. Iodination of the terminal *α*-free pyrrole of **7** followed by a Pd(0)-catalyzed Suzuki–Miyaura cross-coupling reaction with compound **3** gave the desired final receptor **10** in moderate yield (58%). Additionally, compound **10** was prepared by Miyaura borylation of terminal iodo-pyrroles, followed by a Pd(0) cross-coupling reaction with precursor **2** with a similar isolated yield (55%). Compound **10** was characterized by ^1^H-, ^13^C-, and two-dimensional nuclear magnetic resonance (NMR) spectroscopic analyses (correlation spectroscopy (COSY) and nuclear overhauser effect spectroscopy (NOESY)) and mass spectrometry (Figures S9–S12 and S31).

In terms of the characterization of the dimeric receptor (**10**)_2_, we performed electrospray ionization time-of-flight (ESI-TOF) mass spectrometric analysis (Figure S13). The peak at *m*/*z* ≈ 1416 (assigned for [(**10**)_2_ + H]^+^ species) was seen, supporting our proposed dimeric model in the gaseous state. This dimeric form was further confirmed by solid-state single crystallographic analyses (*vide infra*).

### 2.2. X-ray Crystallography

Single crystals of precursor **5** and acyclic receptor **10**, suitable for X-ray crystallographic analyses, were grown from anhydrous CHCl_3_ and anhydrous *N*,*N*-dimethylformamide (DMF) solution by diffusion of hexanes into the respective solutions. The corresponding crystallographic data were uploaded to the Cambridge Crystallographic Data Centre (CCDC) under reference numbers CCDC-2056093, 2056094, and 2058193. As inferred from the X-ray crystallographic analyses, compound **5** possesses a coplanar arrangement that forms an infinite supramolecular polymer of linear “zig-zag” monomers through hydrogen-bonding interactions with adjacent units in the solid state (Figure S34).

**Figure 2:**
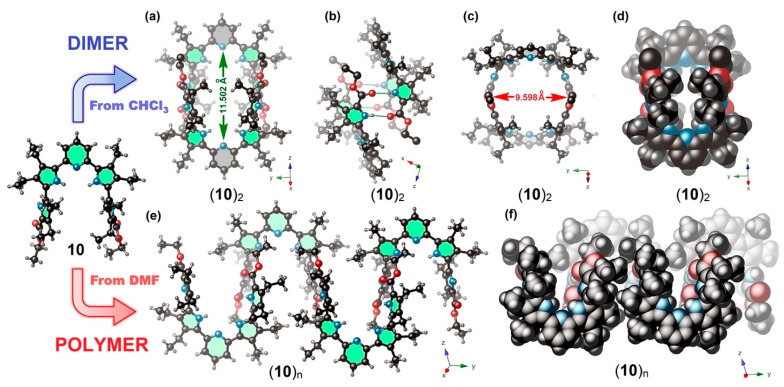
(**a**) Single crystal X-ray structures of receptor **10** grown from two different solvents under ambient conditions. Panels (**a**–**d**) represent a discrete supramolecular dimeric pseudo-macrocyclic cage-like structure of the receptor with the empirical chemical formula (**10**)_2_ grown from anhydrous CHCl_3_. (**e**,**f**) The single-crystal X-ray structure of receptor **10** represents a “zig-zag” 1D hydrogen-bonded supramolecular polymer with empirical chemical formula (**10**)_n_ grown from anhydrous *N*,*N*-dimethylformamide (DMF). For clarity, the solvent molecules (CHCl_3_ and DMF) are omitted from the (**10**)_2_ and (**10**)_n_ crystals, respectively.

As mentioned earlier, receptor **10** preferentially self-assembles in the discrete dimeric form to produce a supramolecular pseudo-macrocyclic cage with the empirical chemical formula (**10**)_2_ when crystalized from a relatively less polar non-coordinating solvent, e.g., CHCl_3_ (Figure 2a–d). This produces a small rectangular inner cavity that is suitable for small acid guests. The “total potential solvent occupied void volume”, which was calculated using PLATON crystallographic software [33], was approximately 1632 Å^3^ (which is 30.9% per unit cell volume, 5289 Å^3^). The crystal packing diagram of (**10**)_2_ is shown in Figure S37a,b. 

**Figure 3:**
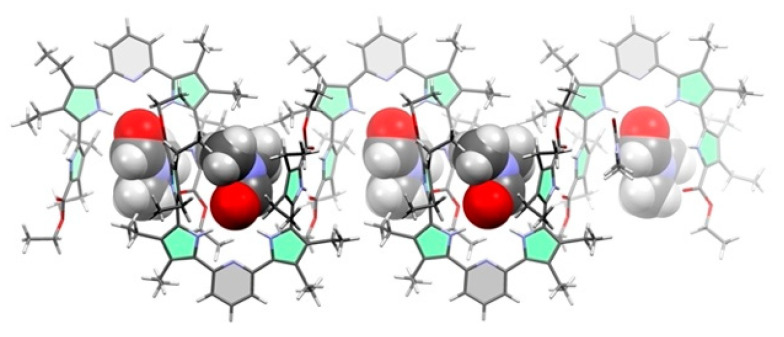
Polar-coordinating solvent-assisted host–guest complex of the hydrogen-bonded 1D supramolecular polymeric structure (DMF)_n_⊂(**10**)_n_ grown from anhydrous DMF. DMF solvent molecules bonded with pyrrolic NH groups are denoted by the space-filling model for clarity.

Conversely, when receptor **10** was crystallized from the polar coordinating solvent *N*,*N*-dimethylformamide (DMF) an infinite 1D supramolecular polymeric structure (Figure 2e,f and Figure S39) was produced by intermolecular hydrogen-bonding interactions. This is believed to be due to the higher coordination capability of DMF molecules (acting as hydrogen bond acceptors) than that of non-coordinating CHCl_3_ molecules. As shown in Figure 3, the DMF solvent molecules are coordinated to the inner pyrrolic NH protons via hydrogen-bonding interactions within the pocket of the “U”-shaped receptor unit. The average N–O bond distance in NH···O=C hydrogen-bonded host–guest complex within the inner pyrroles to the “O” atom of DMF located at the core is ca. 2.892 Å, as seen from X-ray structural analyses (Figure S40). 

The observed solvent-dependent self-assembly in the solid receptor **10** in apolar non-coordinating (where dimeric form predominates) vs. polar coordinating solvent (where the polymeric form predominates) is in stark contrast to our earlier report based on a naphthyridine core (**11**), where only the dimeric unit was observed in solution and solid phase, irrespective of solvent polarity. 

### 2.3. Structural Behavior in Solution

**Figure 4:**
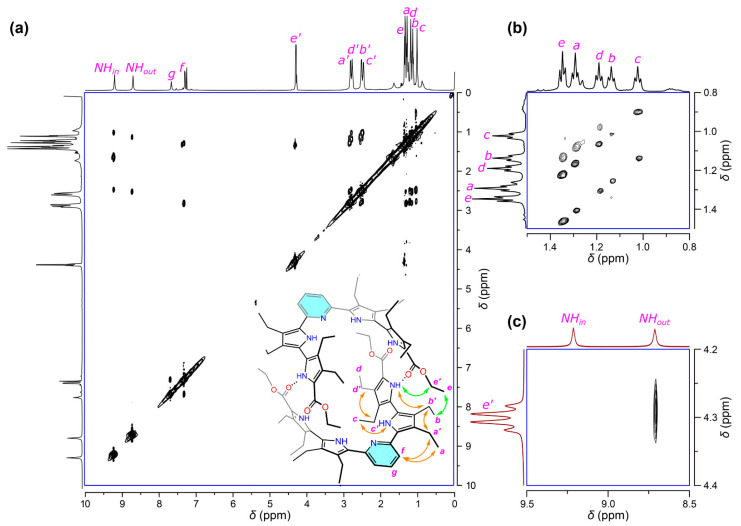
(**a**–**c**) ^1^H–^1^H NOESY spectrum of **10** in CDCl_3_ at 298 K. The inset shows the expected dimer structure with the specific interactions.

In an effort to gain insight into the aggregation properties of **10**, a concentration-dependent ^1^H NMR spectral analysis was performed in CDCl_3_ and THF-*d_8_* (Figure S29a,b). Upon changing the concentration of the host solution from a millimolar (mM) to a micromolar (μM) concentration, no noticeable change in chemical shift was observed (e.g., Δδ = 0.01 ppm for the NH proton resonances at ca. 8.72 and 9.23 ppm in CDCl_3_). Therefore, it was speculated that only a single predominant species was present in the solution phase. Moreover, variable-temperature (VT) ^1^H NMR studies (from 328 to 213 K) in CDCl_3_ revealed a slight change in the chemical shift (Δδ~0.27 ppm) of the signals of pyrrolic NH moieties (δ = 8.72 and 9.23 ppm), indicating weak solvent interactions (Figure S14). Therefore, a robust form of the supramolecular dimeric cage-like structure was present in CDCl_3_. A similar chemical shift distribution pattern was also seen with receptor **11**, adopting a self-assembled dimeric structure [30]. The ^1^H–^1^H NOESY spectrum (Figure 4c) revealed distinct intermolecular spatial interactions between the CH_2_ moiety of the terminal OEt groups and the NH of the pyrrole rings in the receptor, suggesting a dimeric structure in CDCl_3_ at 298 K. In contrast, in relatively high polar and coordinating solvents (e.g., in both THF-*d*_8_ and DMF-*d*_7_), these pyrrolic NH signals shifted significantly (Δδ = 0.85 ppm) upon lowering the temperature, which indicates the considerable solvent coordination of monomeric **10** in these solvents (Figures S15 and S16). Therefore, the dissociated single molecule might be present in these solvents. 

### 2.4. Acid Recognition Studies Using Spectroscopy

To evaluate the capability of receptor **10** for host–guest recognition in solution, ^1^H NMR and absorption spectroscopic analyses were performed through titration of various common organic and mineral acids in CDCl_3_ and THF-*d_8_* at 298 K. During the experiments, a wide range of acids with different p*K*_a_ values ranging from 4.88 (propionic acid) to −6.30 (HCl) were investigated. It was observed that relatively stronger acids (p*K*_a_ ≤ –1.92) were necessary for the initial protonation of the pyridine moiety. After the initial protonation event, subsequent anion binding took place. Introduction of anions as their tetrabutylammonium (TBA^+^) salts did not result in any noticeable change in either ^1^H NMR or absorption studies. We rationalized this observation, as protonation is a primary requirement to achieve the anion binding. Due to the inability of TBA^+^ salts to protonate the pyridine moiety, no change in chemical shift was observed, indicating that there was no complexation taking place. 

**Figure 5:**
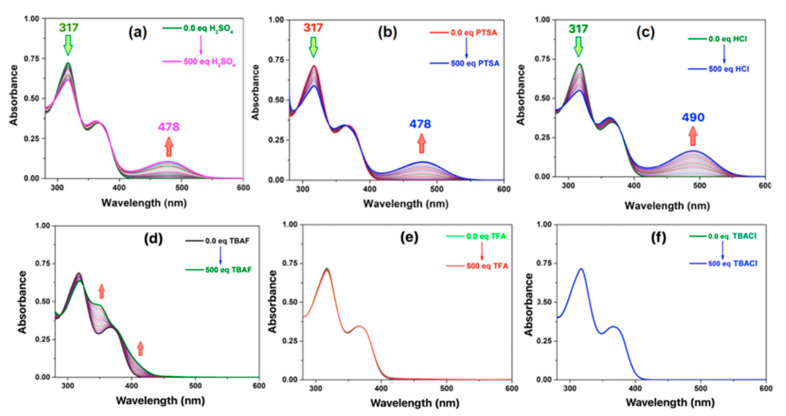
(**a**–**f**) Ultraviolet–visible (UV–vis) spectroscopic titration results showing the changes in the spectral features observed when receptor **10** was titrated against increasing quantities of (up to 500 equiv) H_2_SO_4_, PTSA, HCl, TBAF, TFA, and TBACl, respectively. All of the experiments were carried out with [10] = 2.00 × 10^−5^ M in anhydrous THF at 298 K.

The ultraviolet–visible (UV–vis) spectroscopic titrations were performed with acids in anhydrous THF. All of the controlled titration experiments were carried out by keeping the receptor concentration constant at [10] = 2.00 × 10^−5^ M while varying the titrating acid concentration between 0 and 500 equiv in all the cases, keeping all other experimental conditions identical. Color changes from pale yellow to deep red in the solutions were observed with the naked eye, from which it was found that incremental addition of acid to the receptor induced a hypochromic shift of the absorption maxima at *λ* = 317 nm along with the appearance of a new band at *λ* = 478 nm (in the case of H_2_SO_4_, MSA, and PTSA) and *λ* = 490 nm (in the case of HCl) (Figure 5a–c). These spectral changes were fitted using the multivariate analysis tool BindFit [34,35]. Careful analysis revealed that even after adding up to 500 equiv of these acid guests, there was no indication of the binding event’s reaching saturation. The resulting data were unable to produce any acceptable binding isotherm in BindFit, suggesting very weak binding interactions. No spectroscopic changes were observed after the addition of 500 equiv of weak acids, e.g., acetic acid (Figure S32a), propionic acid (Figure S32b), and TFA (Figure 5e)) and anions in the form of their TBA salt, e.g., TBACl (Figure 5f)), in THF. Furthermore, it was noticed that the peak intensities of the protonated species (λ_max_ = 490 nm for HCl-adduct) could be dependent on the p*K*_a_ of the acids used. Therefore, the most significant change was observed in the case of HCl (which had the lowest value (p*K*_a_ = −6.30) in the series).

**Figure 6:**
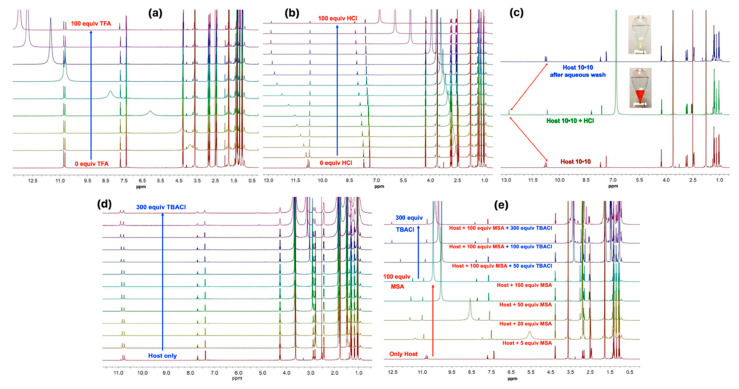
(**a**,**b**) Changes in the ^1^H nuclear magnetic resonance (NMR) spectrum seen when receptor **10** was subjected to titration with increasing quantities (up to 100 equiv) of TFA and HCl in THF-*d_8_* at 298 K. Panel (**c**) demonstrates the reversible guest binding process of **10** with HCl through simple aqueous washing. (Note: the color of the host–guest complex changes from deep red to colorless upon aqueous wash.) (**d**) The ^1^H NMR titration result with the receptor against TBACl in THF-*d*_8_ at 298 K, showing no chemical shift, which indicates that there was no anion binding event. (**e**) Demonstration of the proton-coupled anion binding mechanism involved in the polymeric receptor against strong acids: Protonation via gradual addition of MSA, followed by incremental addition of 300 equiv of TBACl, showing the downfield chemical shift of NH proton signals, indicating an anion binding phenomenon under identical experimental conditions. All of these experiments were carried out with a 5 mM receptor **10** solution in THF-*d_8_* at 298 K.

The ^1^H NMR titrations of **10** ([10] = 5 mM) were performed against incremental addition of 100 equiv of selected acid guests in THF-*d*_8_ at 298 K. There was no discernable change in the proton resonances in ^1^H NMR under identical experimental conditions when receptor **10** was titrated against TFA (p*K*_a_ = 0.23 in water) in THF-*d*_8_, indicating that there was no host–guest binding (Figure 6a). This reflects its inability to protonate the pyridine moiety of receptor **10**. However, when the titrations were performed against MSA (p*K*_a_ = −1.92 in water), PTSA (p*K*_a_ = −2.80 in water), HNO_3_ (p*K*_a_ = −1.3 in water), H_2_SO_4_ (p*K*_a_ = −3.0 in water), and HCl (p*K*_a_ = −6.30 in water), downfield shifts of one of the NH protons (10.75 ppm) were observed (Figures S18–S20). A similar type of spectral signature and the chemical shift were observed when the titration was performed against incremental addition of H_2_SO_4_ guest in DMF-*d_7_* at 298 K (Figure S30). This is a clear indication of the pyrrole NH···anion interactions upon protonation. These phenomena are directly related to the p*K*_a_ of the acids, and the highest chemical shift change (Δ*δ* = 2.25 ppm) was observed in the case of HCl, which is the strongest acid of the series used in this study. Interestingly, the pristine host could be recovered through a simple aqueous wash, as inferred from ^1^H NMR spectroscopic analysis (Figure 6c).

As discussed from the system of receptor **11**, we tested the binding of the organic dicarboxylic acids, such as oxalic acid (p*K*_a1_ = 1.24 in water), maleic acid (p*K*_a1_ = 1.90 in water), malonic acid (p*K*_a1_ = 2.83 in water), fumaric acid (p*K*_a1_ = 3.03 in water), and succinic acid (p*K*_a1_ = 4.20 in water), and monocarboxylic acids, such as acetic acid (p*K*_a_ = 4.76 in water) and propionic acid (p*K*_a_ = 4.88 in water). However, no changes in the chemical shift of the pyrrolic NH signals were observed in the ^1^H NMR spectrum. Likewise, no changes in the absorption maxima were observed in the UV–vis spectroscopic experiments, even after the addition of 500 equiv of these relatively weaker acids. Similarly, this is thought to be due to their relatively weak acidity (p*K*_a_ ≤ −1.92 in water). Therefore, it is speculated that the p*K*_a_ of acids mainly plays an important role during anion recognition within this supramolecular receptor in solution. 

When receptor **10** was titrated using various tetrabutylammonium salts (e.g., TBA^+^Cl^−^, TBA^+^Br^−^, TBA^+^OAc^−^, TBA^+^HSO_4_^−^, etc.) in CDCl_3_, no significant change in chemical shift was observed under identical experimental conditions to those observed above (Figures S27 and S28). Similarly, no chemical shift was observed when the titration was performed in THF-*d_8_* (Figure 6d), indicating that there was no anion binding event. When TBA^+^F^−^ was used, changes in chemical shifts were observed. The addition of TBAF caused deprotonation of the pyrrolic NH protons, as inferred from the ^1^H NMR titration in CDCl_3_ at 298 K (Figure S24). The low-temperature ^1^H NMR spectroscopic analyses revealed the formation of the HF_2_^−^ signal at 16.5 ppm (Figure S25). This change was also observed in the absorption spectra (Figure 5d). These observations led us to assume that protonation is required to achieve the anion binding. 

To confirm this proton-coupled anion binding phenomenon, an additional titration was performed. This has been done through the incremental addition of 100 equiv of MSA to the receptor to protonate the pyridine first, followed by the gradual addition of 300 equiv of TBACl salt as a source of Cl^−^ anions. Interestingly, a downfield chemical shift of the inner NH proton signal was observed upon TBACl addition (Figure 6e). As shown in Figure 6b, there were specific chemical shift changes in the aromatic CH resonances of the pyridine moiety at the para position. The downfield chemical shift of the protons (Δ*δ* = 0.64 ppm, observed in the control experiment of proton-coupled anion binding shown in Figure 6e, and Δ*δ* = 0.51 ppm observed in the case of HCl, Figure 6b) might be the combined effect of protonation-induced change in aromaticity within the central “pyrrole–pyridine–pyrrole” system, followed by an anion binding process. This unequivocally supports our proposed proton-coupled anion binding mechanism involved with this supramolecular receptor.

### 2.5. Theoretical Studies

**Figure 7:**
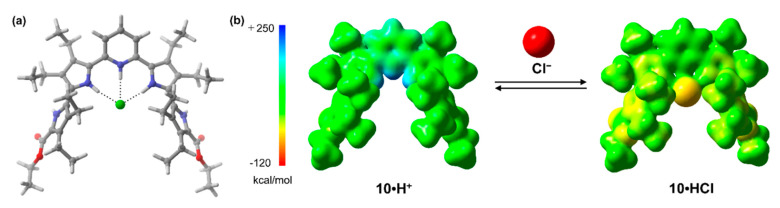
(**a**) Theoretically predicted energy-minimized receptor–acid complex [(**10** + H)^+^(Cl^−^)] in a gas phase. (**b**) The change in the electrostatic potential map (ESP) of the protonated receptor upon anion recognition.

In the host–guest system, hydrogen-bonding interactions are ubiquitous in molecular recognition. The electronic nature and the number of donor sites in the host molecules vary the complementary geometries of the complexes. Therefore, to gain structural insight into the binding mode of the host–guest complexes, we performed theoretical calculations by employing density functional theory (DFT) in the gas phase. M06-X2 calculations yielded a predicted protonated complex [(**10** + H)^+^(Cl^−^)] with HCl as an acid (Figure 7a). The protonation of the pyridine moiety in **10** offers a specific cavity surrounded by hydrogen-bonding donors, stabilizing a chloride-bound complex with the estimated gas-phase binding energy of −17.9 kcal mol^−^^1^. In the comparative analysis of the electrostatic potential (ESP) map of **10** and the 1,8-naphthyridine-analogue **11** reported earlier [30], a greater extent of negative potential around the naphthyridine-nitrogens was observed, indicating a higher basicity (Figure S41). Even considering the intrinsically high basicity of the pyridine ring, the cavity is relatively crowded by adjacent pyrrole rings and *β*-ethyl groups, which may hamper the protonation at the pyridine moiety in **10**. The outer pyrroles attached to the terminal ester groups in (**10**)_2_ contribute solely towards dimer formation with a neighboring unit. It is speculated that in the polymeric unit of receptor **10**, HCl also binds inside the pocket. The expected supramolecular dimeric complex, [(**10** + H)^+^(Cl^−^)]_2_ in the gas phase, was also estimated to be −62.5 kcal mol^−1^ (Figure S42). As observed for the anion-exchange experiment, the positive electrostatic potential (ESP) at the cavity of the protonated **10** (i.e., **10** + H^+^) leads to a reduction in the negative ESP of the chloride anion (Figure 7b). 

## 3. Materials and Methods

### 3.1. General Experiments 

#### 3.1.1. Chemicals and Consumables 

All the chemicals used during the synthesis of receptor **10** were purchased from various commercial sources and directly used without further purification.

- Iodine (I_2_) (CAS No: 7553-56-2), Triethylamine (Et_3_N) (CAS No: 121-44-8), Potassium carbonate (K_2_CO_3_) (CAS No: 584-08-7), Sodium bicarbonate (NaHCO_3_) (CAS No: 144-55-8), Sodium thiosulfate (Na_2_S_2_O_3_) (CAS No: 7772-98-7), Dichloromethane (CH_2_Cl_2_) (CAS No: 75-09-2) were purchased from Adamas-beta^®^, Shanghai, China.
- Sodium hydroxide (NaOH) (CAS No: 1310-73-2), Ethanol (EtOH) (CAS No: 64-17-5) were purchased from Energy Chemical, Shanghai, China.
- Palladium(II) acetate, Pd(OAc)_2_ (CAS No: 3375-31-3), Tetrakis(triphenylphosphine)palladium, Pd(PPh_3_)_4_ (CAS No: 14221-01-3), Triphenylphosphine, PPh_3_ (CAS No: 603-35-0), Dichlorobis(triphenylphosphine)palladium(II) (PdCl_2_(PPh_3_)_2_) (CAS No: 13965-03-2) were purchased from Merck-Sigma, Shanghai, China.
- Potassium iodide (KI) (CAS No: 7681-11-0), *N*,*N*-Dimethylformamide (DMF) (CAS No: 68-12-2), Ethylene glycol (EG) (CAS No: 107-21-1), 1,4-Dioxane (CAS No: 123-91-1) were purchased from Acros Organics, NJ, USA.
- Pinacolborane, HB(pin) (CAS No: 25015-63-8), 2,6-Dibromopyridine (CAS No: 626-05-1) were purchased from TCI, Tokyo, Japan.
- All anhydrous solvents were either purchased from Sigma Aldrich, Shanghai, China or Acros Organics Shanghai China or collected from the solvent purification plant at the Center for Supramolecular Chemistry and Catalyses, Shanghai University. All reactions were carried out under an argon atmosphere unless noted otherwise.
- Chromatographic separations were performed by using 100–200 mesh silica gel obtained from Merck-Sigma, Darmstadt, Germany.
- The thin-layer chromatographic (TLC) analyses were carried out using Silica Gel 60 F_245_ glass sheets, which were used to monitor the progress of the reactions and were purchased from Merck-Sigma, Darmstadt, Germany.
- Final separations of the compounds were performed using a recycling preparative gel permeation chromatography (GPC) from Japan Analytical Industries (JAI), Hiroshima Japan using THF as the mobile phase. The oligopyrrole derivatives **1**–**9** were synthesized with moderate to good yields by following the procedures in the literature [30,31,32].

#### 3.1.2. Investigation and Characterization 

^1^H NMR (600 MHz) and ^13^C NMR (150 MHz) data were collected at room temperature on a Bruker 600 MHz NMR spectrometer using CDCl_3_, THF-*d*_8_, or DMF-*d*_7_ at 298 K unless noted otherwise. All chemical shifts (^1^H NMR and ^13^C NMR ppm) were reported in (*δ*, ppm) relative to the residual deuterated solvent peaks of CDCl_3_ (7.25 ppm) or CD_2_Cl_2_ (5.32 ppm). MALDI-TOF mass spectrometric measurements were made using a Bruker (Autoflex speed) matrix-assisted laser desorption ionization time-of-flight mass spectrometer. Some compounds were analyzed using a Bruker Microflex 2 LRF 20 spectrometer using dithranol (1,8,9-trihydroxyanthracene) as the matrix. UV–vis spectra were recorded in anhydrous THF at 298 K using a Varian Cary 50 spectrophotometer. The concentration of all the entities was maintained at 2 × 10^−5^ M in THF. 

### 3.2. Synthesis

#### Synthesis of Acyclic Receptor **10**

A mixture of **8** (0.5 mmol), Pd(PPh_3_)_4_ (0.025 mmol), and K_2_CO_3_ (2.5 mmol) was suspended in a mixture of DMF (10 mL) and water (5 mL), and the reaction was heated to 95 °C under an Ar atmosphere. Compound **3** (1.2 mmol) was dissolved in DMF (4 mL) and added via a syringe pump over the course of 1 h. The reaction was stirred at the same temperature for another 15 h. After the reaction mixture was cooled to room temperature, water was added. The organic layer was extracted with CH_2_Cl_2_ and washed with water three times. After removing the solvent, the reaction mixture was purified through silica gel chromatography using petroleum ether/ethyl acetate (20/1 to 10/1, (*v*/*v*)) as eluent to get **10** as a light-yellow solid in 55% yield. Another way to get the poly-pyrrolic receptor **10** was synthesized through the Pd(II)-catalyzed Suzuki–Miyaura cross-coupling reaction between precursors **9** and **2** to obtain **10** in 58% yield. ^1^H NMR (600 MHz, CDCl_3_): *δ*: 9.23 (s, 2H, pyrrole NH), 8.72 (s, 2H, pyrrole NH), 7.67 (t, *J* = 7.9 Hz, 1H, pyridine CH), 7.30 (d, *J* = 7.9 Hz, 2H, pyridine CH), 4.29 (q, *J* = 7.1 Hz, 4H, CH_2_), 2.81 (q, *J* = 7.3 Hz, 4H, pyrrole CH_2_), 2.76 (q, *J* = 7.3 Hz, 4H, pyrrole CH_2_), 2.52 (q, *J* = 7.4 Hz, 4H, pyrrole CH_2_), 2.46 (q, *J* = 7.4 Hz, 4H, pyrrole CH_2_), 1.34 (t, *J* = 7.1 Hz, 6H, pyrrole CH_3_), 1.29 (t, *J* = 7.5 Hz, 6H, pyrrole CH_3_), 1.18 (t, *J* = 7.4 Hz, 6H, pyrrole CH_3_), 1.13 (t, *J* = 7.5 Hz, 6H, pyrrole CH_3_), 1.01 (t, *J* = 7.4 Hz, 6H, CH_3_) ppm. ^13^C NMR (150 MHz, CDCl_3_): *δ*: 161.26, 149.81, 137.26, 133.54, 127.02, 126.17, 125.24, 125.09, 125.03, 120.67, 118.09, 115.46, 59.84, 18.51, 18.34, 17.77, 17.73, 16.56, 16.33, 15.75, 15.41, 14.46 ppm. MALDI-TOF MS: Calcd for C_43_H_57_N_5_O_4_, 707.960. Found: 708.364 [M + H]^+^.

### 3.3. Theoretical Calculations

All calculations were performed using the Gaussian16 program package [36]. The meta-hybrid functional M06-2X was employed for the DFT calculations, and the 6-31G(d) basis set was employed for all atoms [37]. The crystal structure of **10** was used as an input file for generating the supramolecular complex with HCl. All calculated geometries were checked to be true minima through vibrational analysis and showed no imaginary frequencies. According to Equation (1), the free energies (Δ*G*) of the monomers (hosts and acids separately) and the corresponding complexes were calculated. They include standard-state zero-point energies (Δ*E*) and thermal corrections (Δ*G*_T_). The association free energy of each host–guest system was calculated according to Equation (2), which includes the basis set superposition error (BSSE) determined by counterpoise correction [38,39]. All graphics on optimized structures were generated with CYLview [40].

Δ*G* = Δ*E* + Δ*G*_T_ (1)

Δ*G*_BE_ = *G*(complex) − *G*(host) − *G*(guest)+ (2)

## 4. Conclusions 

A fully organic “U”-shaped acyclic pyridine-tetrapyrrolic receptor was designed and synthesized. This receptor self-assembles as a robust dimeric pseudo-macrocyclic cage from a nonpolar, non-coordinating solvent. However, it forms a 1D “zig-zag” supramolecular polymeric chain in polar coordinating solvents. The structures of cage-like dimeric form and polymeric self-assembled constructs were confirmed through single-crystal X-ray diffraction analyses. The pyridine moiety within the receptor in our current system **10** promotes the recognition of strong acids within the cavity. Host–guest complexation studies using various organic and mineral acids were performed with ^1^H NMR and UV–vis spectroscopic analyses. The receptor acts as a potential sensor for strong (both organic and mineral) acids with a p*K*_a_ value < −1.92 in water, with a visible color change from pale yellow to deep red. The pure receptor can be easily recovered from the organic host–guest mixture after simple aqueous washing. Detailed experimental analyses of these supramolecular systems suggest a two-step proton-coupled anion recognition process. This complete recognition study of an acyclic oligopyrrole in the solution phase leads us to suggest that the pyridine-appended oligopyrrole might be a promising candidate for the “capture and release” of strong acids from laboratory or industrial wastes by employing the liquid–liquid extraction technique. 

## Data Availability

The data presented in this study are available on request from the corresponding author.

## Supplementary Materials

The following are available online, ^1^H and ^13^C NMR and Mass spectral analyses, additional photophysical data from UV-vis spectroscopic analyses, single crystal X-ray crystallographic analyses and the tables with all the crystallographic data and structure refinement parameters for all the crystals (**5** and **10**), and Cartesian coordinates for receptors **10**, **11**, [**10**H^+^•Cl^−^].

## Sample Availability

Samples of all the synthesized compounds are available from F.W.

## References

[1] Hof F., Rebek J.J. (2002). Molecules within molecules: Recognition through self-assembly. Proc. Natl. Acad. Sci. USA.

[2] Ebbing M.H., Villa M.J., Valpuesta J.M., Prados P., de Mendoza J. (2002). Resorcinarenes with 2-benzimidazolone bridg-ES: Self-aggregation, self-assembled dimeric capsules, and guest encapsulation. Proc. Natl. Acad. Sci. USA.

[3] Ajami D., Rebek J. (2013). More chemistry in small space. Acc. Chem. Res..

[4] Wu N.-W., Rebek J.J. (2016). Cavitands as chaperones for monofunctional and ring-forming reactions in water. J. Am. Chem. Soc..

[5] Tian J., Zhang Y., Du L., He Y., Jin X.-H., Pearce S., Eloi J.-C., Harniman R.L., Alibhai D., Ye R. (2020). Tailored self-assembled photocatalytic nanofibres for visible-light-driven hydrogen production. Nat. Chem..

[6] Fujita D., Suzuki K., Sato S., Yagi-Utsumi M., Yamaguchi Y., Mizuno N., Kumasaka T., Takata M., Noda M., Uchiyama S. (2012). Protein encapsulation within synthetic molecular hosts. Nat. Commun..

[7] Schmidt A., Molano V., Hollering M., Pöthig A., Casini A., Kühn F.E. (2016). Evaluation of new palladium cages as potential delivery systems for the anticancer drug cisplatin. Chem. A Eur. J..

[8] Casini A., Woods B., Wenzel M. (2017). The promise of self-assembled 3D supramolecular coordination complexes for biomedical applications. Inorg. Chem..

[9] Chen J., Zhang Y., Meng Z., Guo L., Yuan X., Zhang Y., Chai Y., Sessler J.L., Meng Q., Li C. (2020). Supramolecular combination chemotherapy: A PH-responsive co-encapsulation drug delivery system. Chem. Sci..

[10] He Q., Tu P., Sessler J.L. (2018). Supramolecular chemistry of anionic dimers, trimers, tetramers, and clusters. Chem.

[11] Peng S., He Q., Vargas-Zúñiga G.I., Qin L., Hwang I., Kim S.K., Heo N.J., Lee C.-H., Dutta R., Sessler J.L. (2020). Strapped Calix[4]pyrroles: From synthesis to applications. Chem. Soc. Rev..

[12] Xia D., Wang P., Ji X., Khashab N.M., Sessler J.L., Huang F. (2020). Functional supramolecular polymeric networks: The marriage of covalent polymers and macrocycle-based host–guest interactions. Chem. Rev..

[13] Wyler R., De Mendoza J., Rebek J. (1993). A synthetic cavity assembles through self-complementary hydrogen bonds. Angew. Chem. Int. Ed..

[14] Ajami D., Rebek J. (2007). Longer guests drive the reversible assembly of hyperextended capsules. Angew. Chem. Int. Ed..

[15] Zhang K.-D., Ajami D., Rebek J. (2013). Hydrogen-bonded capsules in water. J. Am. Chem. Soc..

[16] Fujita M. (1998). Metal-directed self-assembly of two- and three-dimensional synthetic receptors. Chem. Soc. Rev..

[17] Leininger S., Olenyuk B., Stang P.J. (2000). Self-assembly of discrete cyclic nanostructures mediated by transition met-als. Chem. Rev..

[18] Dalgarno S.J., Power N.P., Atwood J.L. (2008). Metallo-supramolecular capsules. Co-ord. Chem. Rev..

[19] Zhang H., Lee J., Brewster J.T., Chi X., Lynch V.M., Sessler J.L. (2019). Cation-based structural tuning of pyridine dipyrrolate cages and morphological control over their self-assembly. J. Am. Chem. Soc..

[20] Kusukawa T., Fujita M. (1999). “Ship-in-a-bottle” formation of stable hydrophobic dimers of cis-azobenzene and -stilbene derivatives in a self-assembled coordination nanocage. J. Am. Chem. Soc..

[21] Yoshizawa M., Takeyama Y., Kusukawa T., Fujita M. (2002). Cacity-directed, highly stereoselective [2+2] photodimeri-zation of olefins within self-assembled coordination cages. Angew. Chem. Int. Ed..

[22] Ueda Y., Ito H., Fujita D., Fujita M. (2017). Permeable self-assembled molecular containers for catalyst isolation ena-bling two-step cascade reactions. J. Am. Chem. Soc..

[23] Chi X., Peters G.M., Hammel F., Brockman C., Sessler J.L. (2017). Molecular recognition under interfacial conditions: Calix[4]pyrrole-based cross-linkable micelles for ion pair extraction. J. Am. Chem. Soc..

[24] Ji X., Chi X., Ahmed M., Long L., Sessler J.L. (2019). Soft materials constructed using calix[4]pyrrole- and “Texas-sized” box-based anion receptors. Acc. Chem. Res..

[25] He Q., Vargas-Zúñiga G.I., Kim S.H., Kim S.K., Sessler J.L. (2019). Macrocycles as Ion pair receptors. Chem. Rev..

[26] Ikeda T., Hirata O., Takeuchi M., Shinkai S. (2006). Highly enantioselective recognition of dicarboxylic acid substrates by the control of nonlinear responses. J. Am. Chem. Soc..

[27] Engle J.M., Lakshminarayanan P.S., Carroll C.N., Zakharov L.N., Haley M.M., Johnson D.W. (2011). Molecular self-assembly: Solvent guests tune the conformation of a series of 2,6-bis(2-anilinoethynyl)pyridine-based ureas. Cryst. Growth Des..

[28] Zhang Z., Kim D.S., Lin C.-Y., Zhang H., Lammer A.D., Lynch V.M., Popov I., Miljanić O.Š., Anslyn E.V., Sessler J.L. (2015). Expanded porphyrin-anion supramolecular assemblies: Environmentally responsive sensors for organic SOL-vents and anions. J. Am. Chem. Soc..

[29] Vonnegut C.L., Shonkwiler A.M., Zakharov L.N., Haley M.M., Johnson D.W. (2016). Harnessing solid-state packing for selective detection of chloride in a macrocyclic anionophore. Chem. Commun..

[30] Wang F., Sen S., Chen C., Bähring S., Lei C., Duan Z., Zhang Z., Sessler J.L., Jana A. (2019). Self-assembled cagelike receptor that binds biologically relevant dicarboxylic acids via proton-coupled anion recognition. J. Am. Chem. Soc..

[31] Setsune J.-I., Toda M., Watanabe K., Panda P.K., Yoshida T. (2006). Synthesis of bis(pyrrol-2-yl)arenes by Pd-catalyzed cross coupling. Tetrahedron Lett..

[32] Zhang Z., Lim J.M., Ishida M., Roznyatovskiy V.V., Lynch V.M., Gong H.Y., Yang X., Kim D., Sessler J.L. (2012). Cy-clo[m]Pyridine[n]Pyrroles: Hybrid macrocycles that display expanded π-conjugation upon protonation. J. Am. Chem. Soc..

[33] Spek A.L. (2015). Platon squeeze: A tool for the calculation of the disordered solvent contribution to the calculated structure factors. Acta Crystallogr. Sect. C Struct. Chem..

[34] Bindfit-Fit Data to 1:1, 1:2 and 2:1 Host-Guest Equilibria. http://supramolecular.org.

[35] Thordarson P. (2011). Determining association constants from titration experiments in supremolecular chemistry. Chem. Soc. Rev..

[36] Frisch M.J., Trucks G.W., Schlegel H.B., Scuseria G.E., Robb M.A., Cheeseman J.R., Scalmani G., Barone V., Petersson G.A., Nakatsuji H. (2016). Gaussian 16, Revision, C.01.

[37] Zhao Y., Truhlar D.G. (2008). The M06 suite of density functionals for main group thermochemistry, thermochemical kinetics, noncovalent interactions, excited states, and transition elements: Two new functionals and systematic testing of four M06-class functionals and 12 other functionals. Theor. Chem. Acc..

[38] Boys S.F., Bernardi F. (1970). The calculation of small molecular interaction by the differences of separate total energies. Some procedures with reduced errors. Mol. Phys..

[39] Turi L., Dannenberg J.J. (1993). Correcting for basis set superposition error in aggregates containing more than two molecules: Ambiguities in the calculation of the counterpoise correction. J. Phys. Chem..

[40] Legault C.Y. (2009). CYLview, 1.0 b.

