# Supplementary material for: Solvent-Controlled Self-Assembled Oligopyrrolic Receptor
Source: Molecules. 2021 Mar 22;26(6):1771. doi: 10.3390/molecules26061771 (PMC8004224; doi:10.3390/molecules26061771)
Supplement: Supplementary file 1 [file molecules-26-01771-s001.pdf]

Supplementary Information

Solvent-controlled self-assembled oligopyrrolic receptor

Fei Wang[1], Kejiang Liang[1], Mads Christian Larsen[2], Steffen Bähring[2,*], Masatoshi Ishida[3,*], Hiroyuki Furuta[3,*] and Atanu Jana[4,*]

[1]Center for Supramolecular Chemistry and Catalysis, Department of Chemistry, Shanghai University, No 333 Nanchen Road, Baoshan District, Shanghai, 200444, P. R. China.

[2]Department of Physics, Chemistry and Pharmacy, University of Southern Denmark, Campusvej 55, 5230, Odense M, Denmark; E-mail: sbahring@sdu.dk (SB)

[3]Department of Chemistry and Biochemistry, Graduate School of Engineering, and Center for Molecular Systems, Kyushu University, Fukuoka 819-0395, Japan; E-mails: ishida.masatoshi.686@m.kyushu-u.ac.jp (MI); hfuruta@cstf.kyushu-u.ac.jp (HF)

[4]Department of Chemistry, Gandhi Institute of Technology and Management (GITAM), NH 207, Nagadenehalli, Doddaballapur Taluk, Bengaluru – 561203, Karnataka, India. E-mail: atanu.j78@gmail.com, ajana2@gitam.edu (AJ)

Table of Contents

I. NMR & Mass Spectral Analyses.. S2-S15
II. Additional Photophysical Data from UV−vis Spectroscopic Analyses....................................S15
III. Single Crystal X-ray Crystallography...S15-S22
IV. Additional Data from DFT Calculations..S22
V. References & Notes..S23
VI. Cartesian Coordinates………………………………………………………..………………… S23-S35

I. NMR & Mass Spectral Analyses

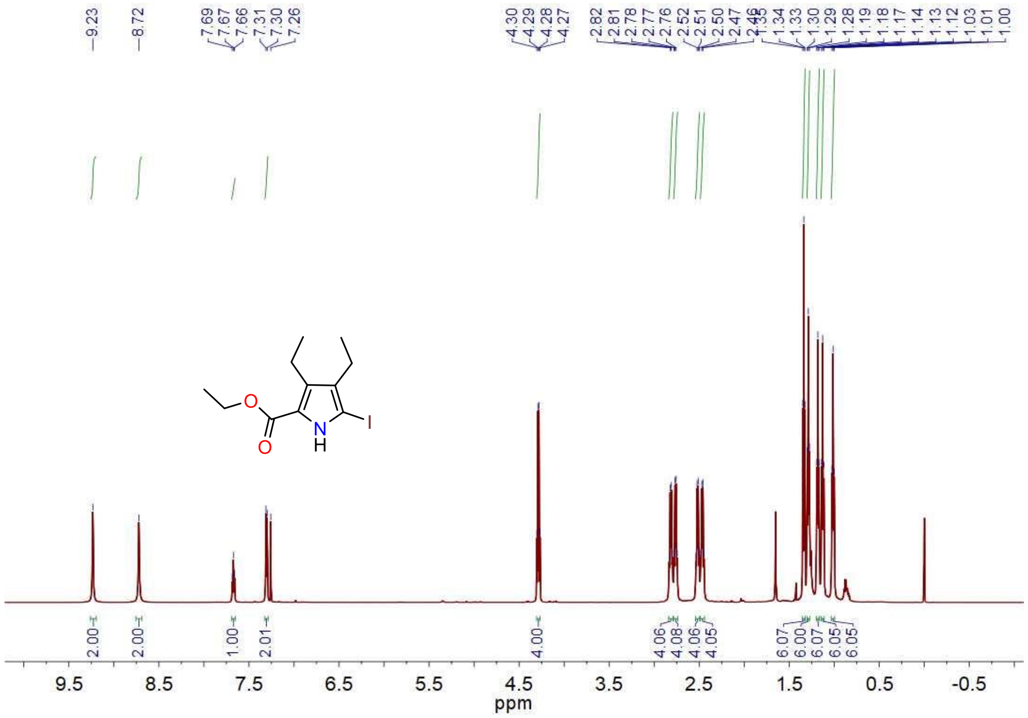

Figure S1. ^{1}H NMR spectrum of **2** recorded in $CDCl_3$ at 298 K.

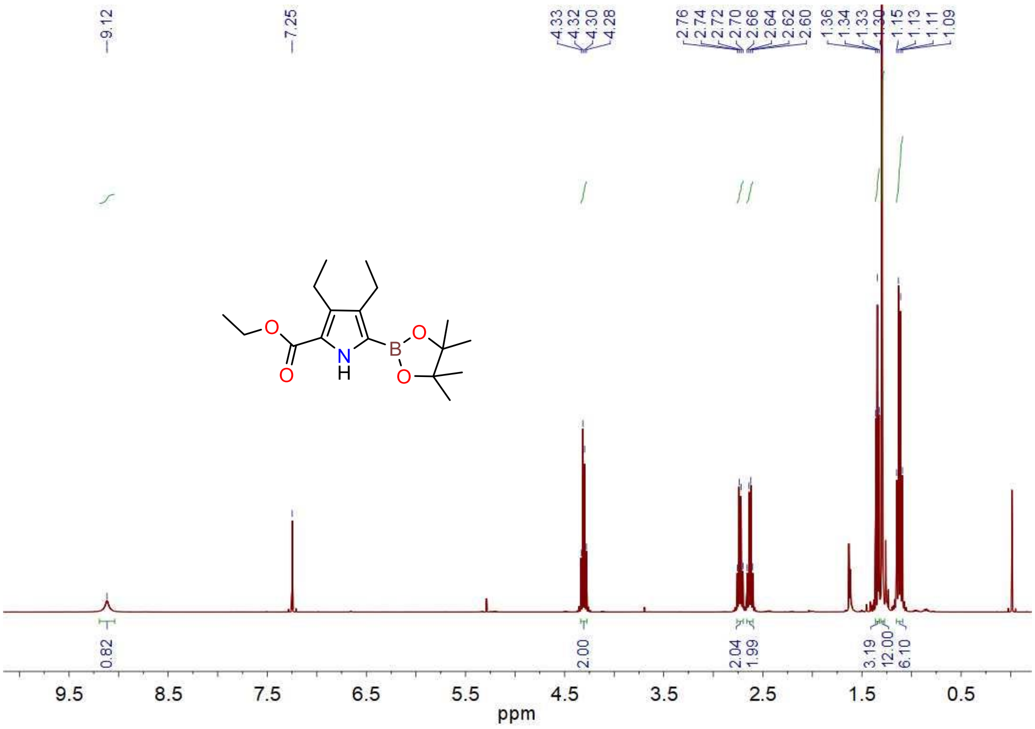

Figure S2. ^{1}H NMR spectrum of **3** recorded in $CDCl_3$ at 298 K.

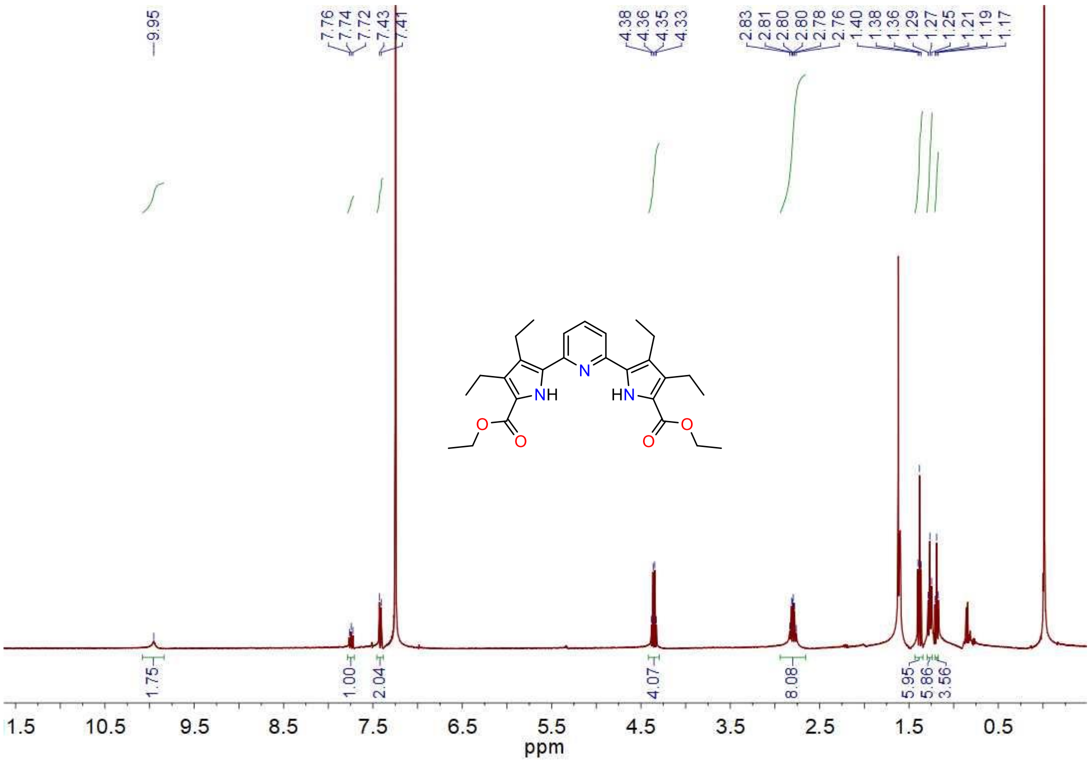

Figure S3. ^{1}H NMR spectrum of **5** recorded in $CDCl_3$ at 298 K.

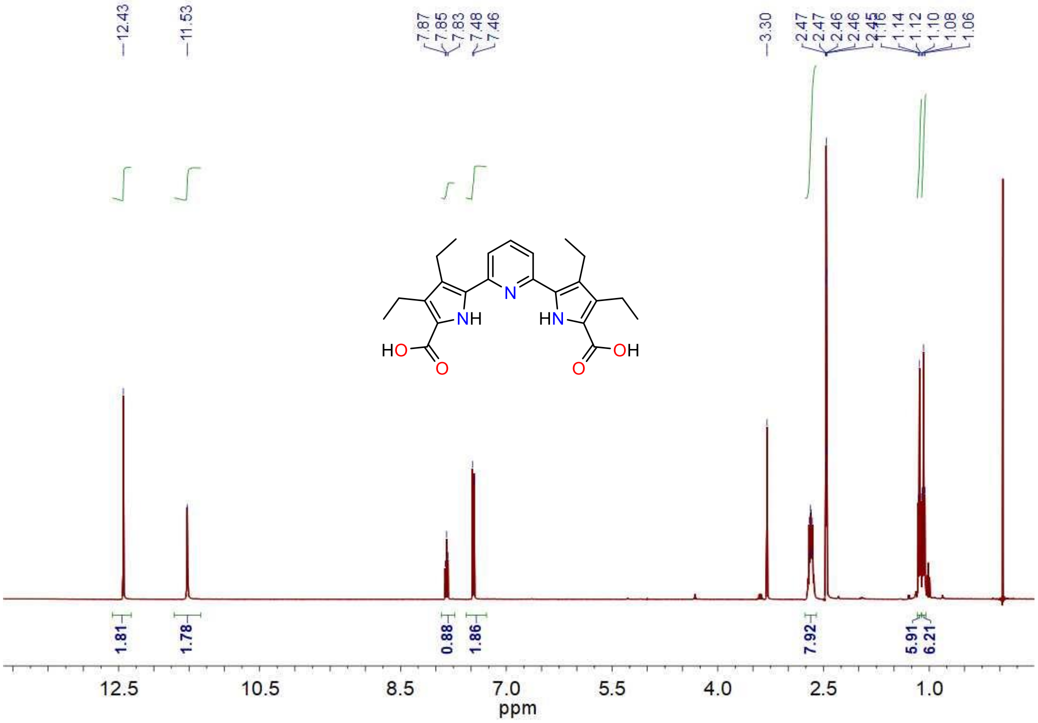

Figure S4. ^{1}H NMR spectrum of **6** recorded in DMSO-d_6 at 298 K.

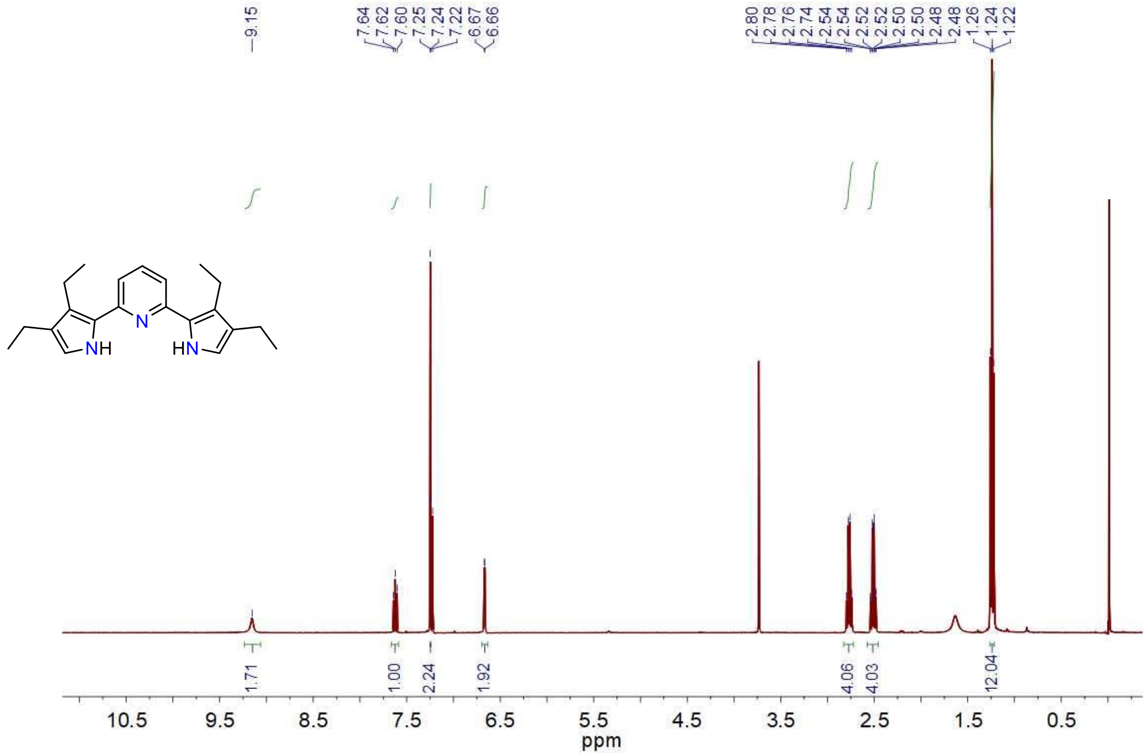

Figure S5. ^{1}H NMR spectrum of **7** recorded in $CDCl_3$ at 298 K.

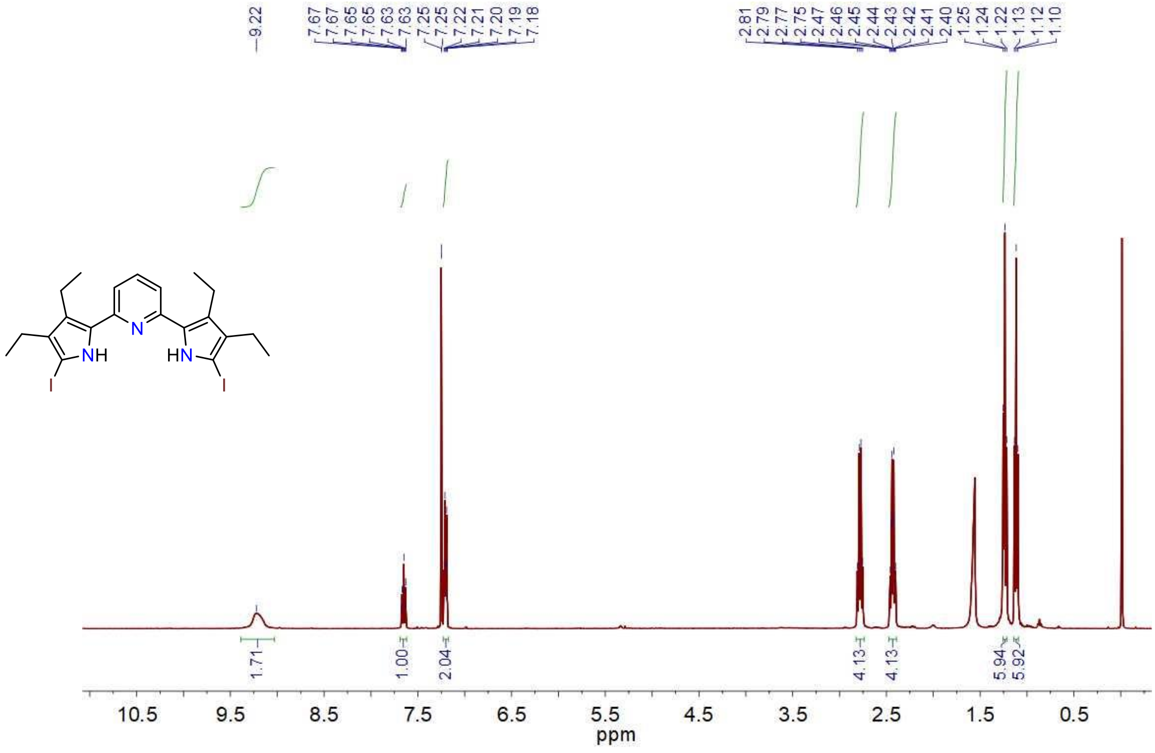

Figure S6. ^{1}H NMR spectrum of **8** recorded in $CDCl_3$ at 298 K.

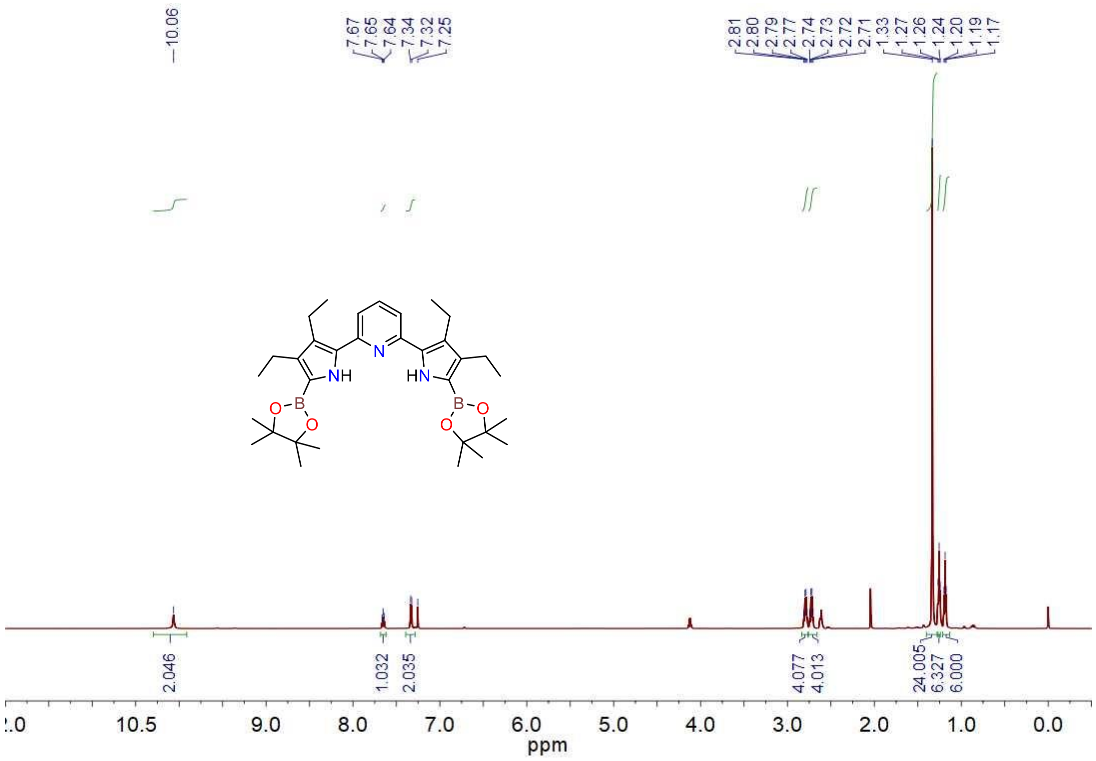

Figure S7. ^{1}H NMR spectrum of **9** recorded in $CDCl_3$ at 298 K.

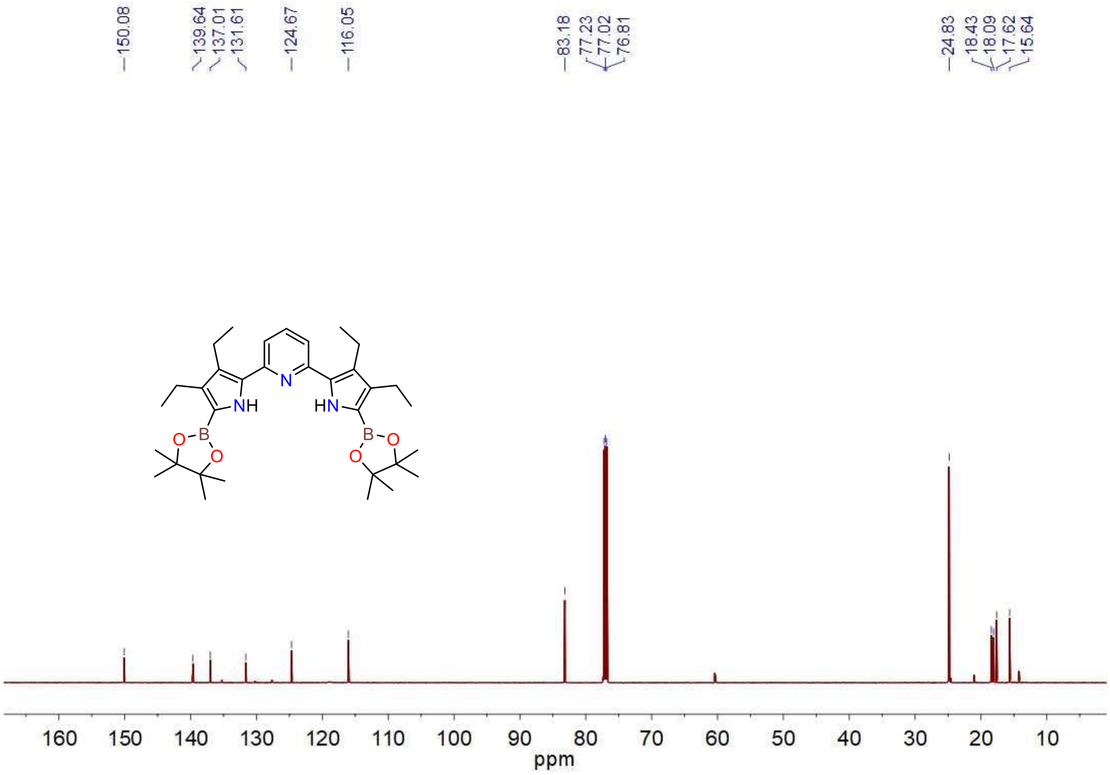

Figure S8. ^{13}C NMR spectrum of **9** recorded in $CDCl_3$ at 298 K.

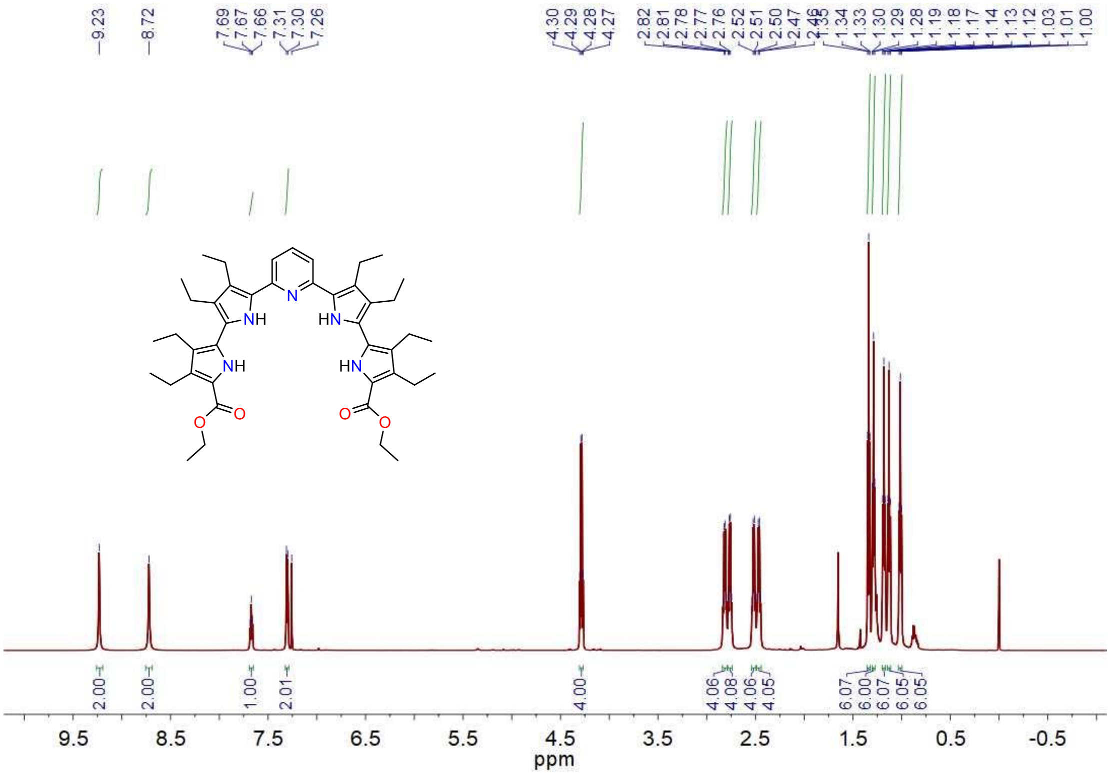

Figure S9. ^{1}H NMR spectrum of receptor **10** recorded in $CDCl_3$ at 298 K.

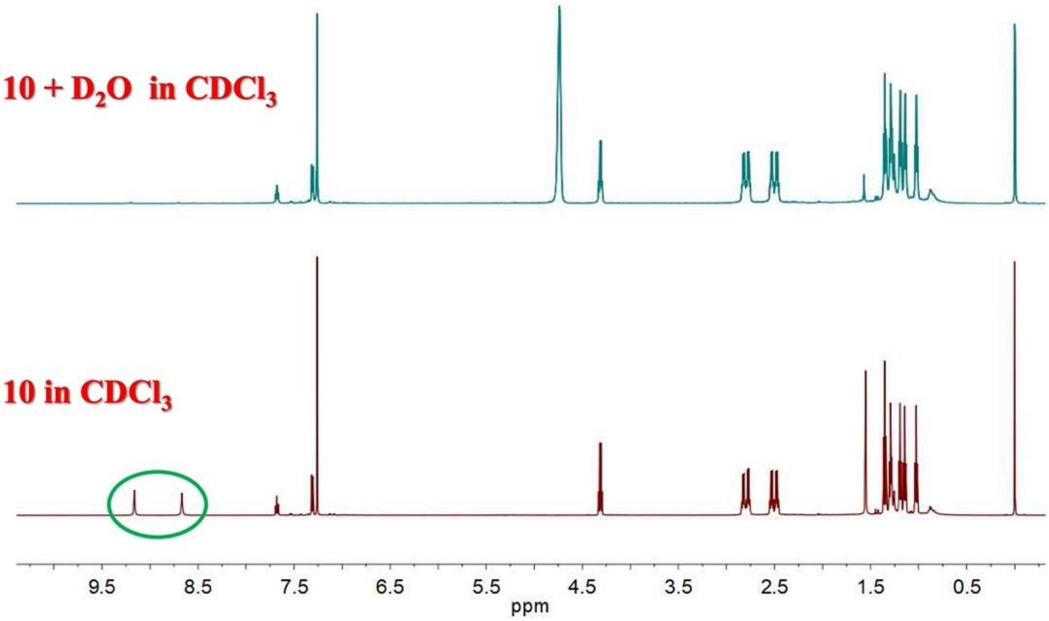

Figure S10. ^{1}H NMR spectra of **10** (bottom) and **10** in presence of D_2O (top) recorded in $CDCl_3$ at 298 K.

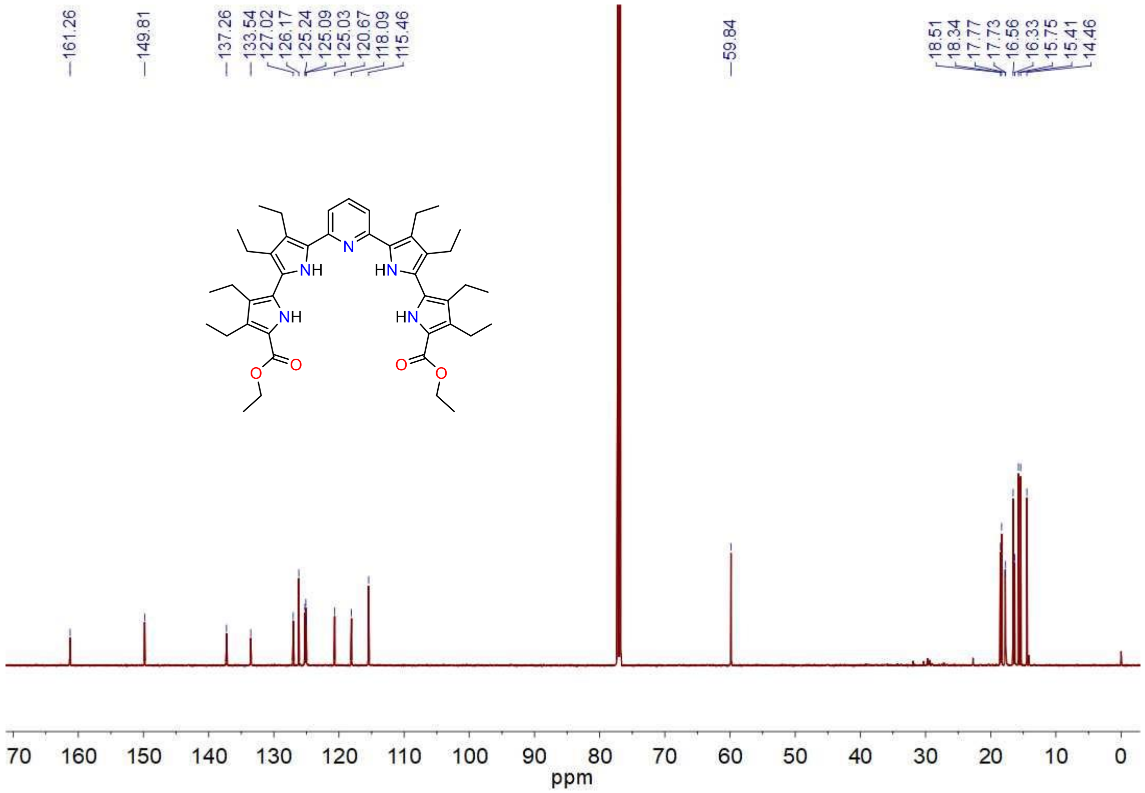

Figure S11. ^{13}C NMR spectrum of receptor **10** recorded in $CDCl_3$ at 298 K.

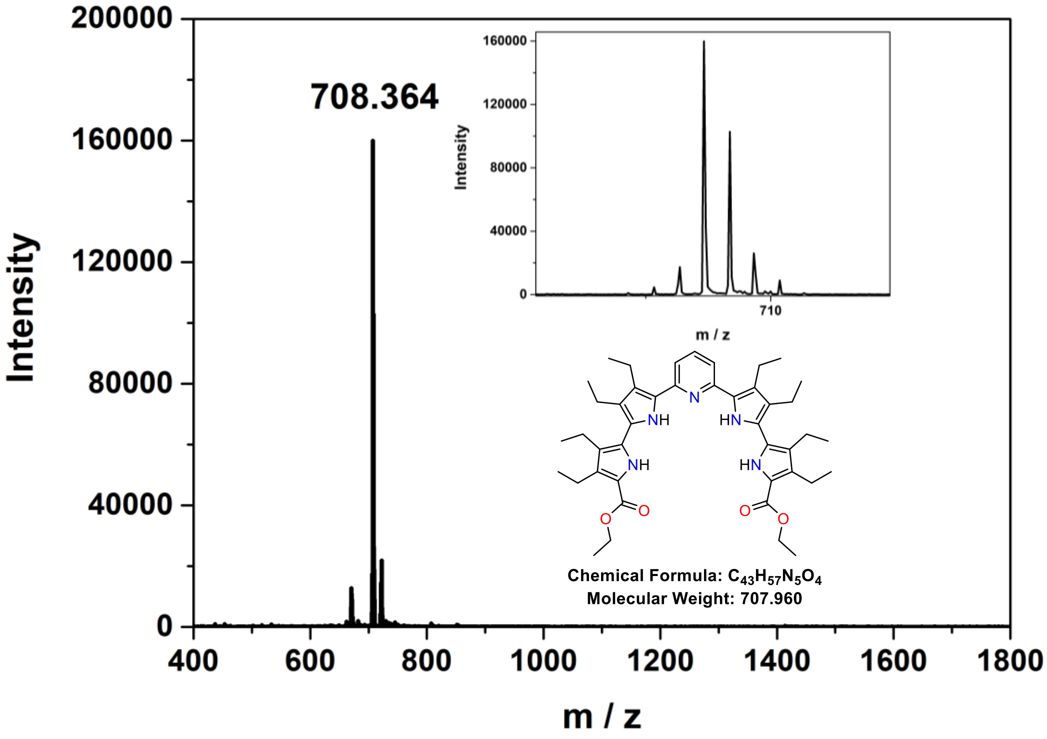

Figure S12. MALDI-TOF mass spectrum of receptor **10**.

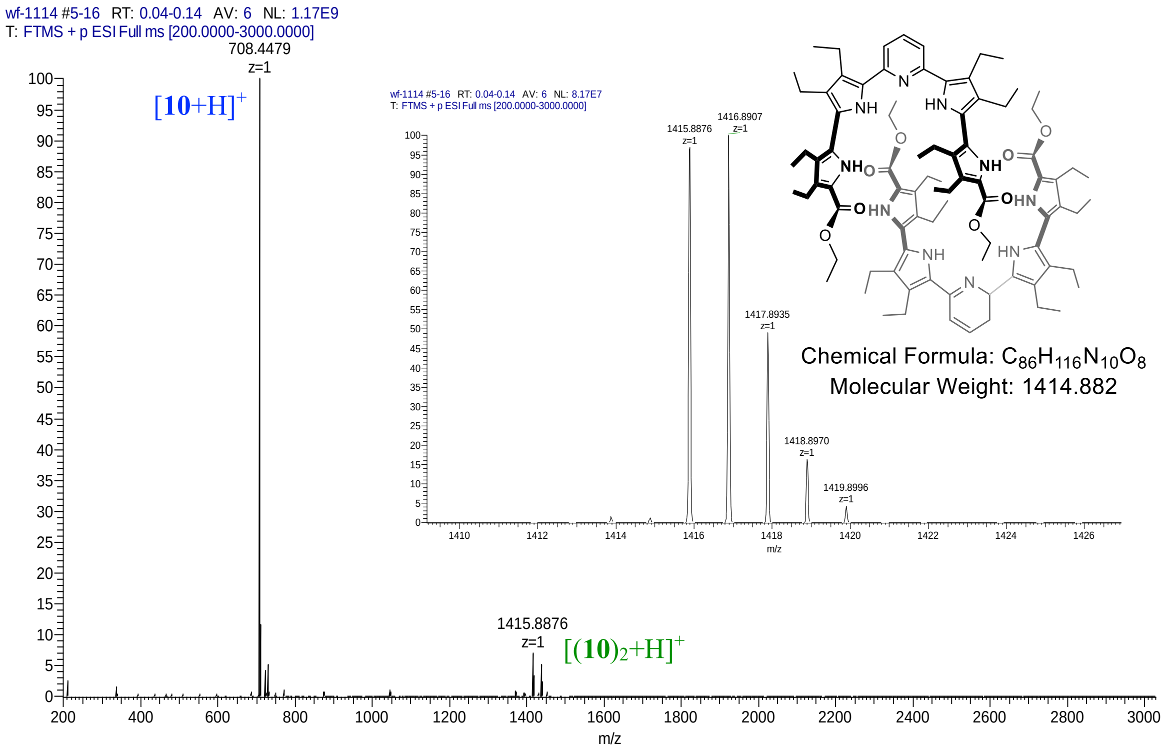

Figure S13. ESI-TOF MS of the receptor **10**, showing ‘m/z’ corresponding to the hydrogen-bonded dimeric macrocyclic species (**10**)$_2$. **Inset**: Isotopic distribution pattern of the dimeric macrocyclic receptor (**10**)$_2$ predicted by ESI-TOF MS, considering $(M+1)^+$.

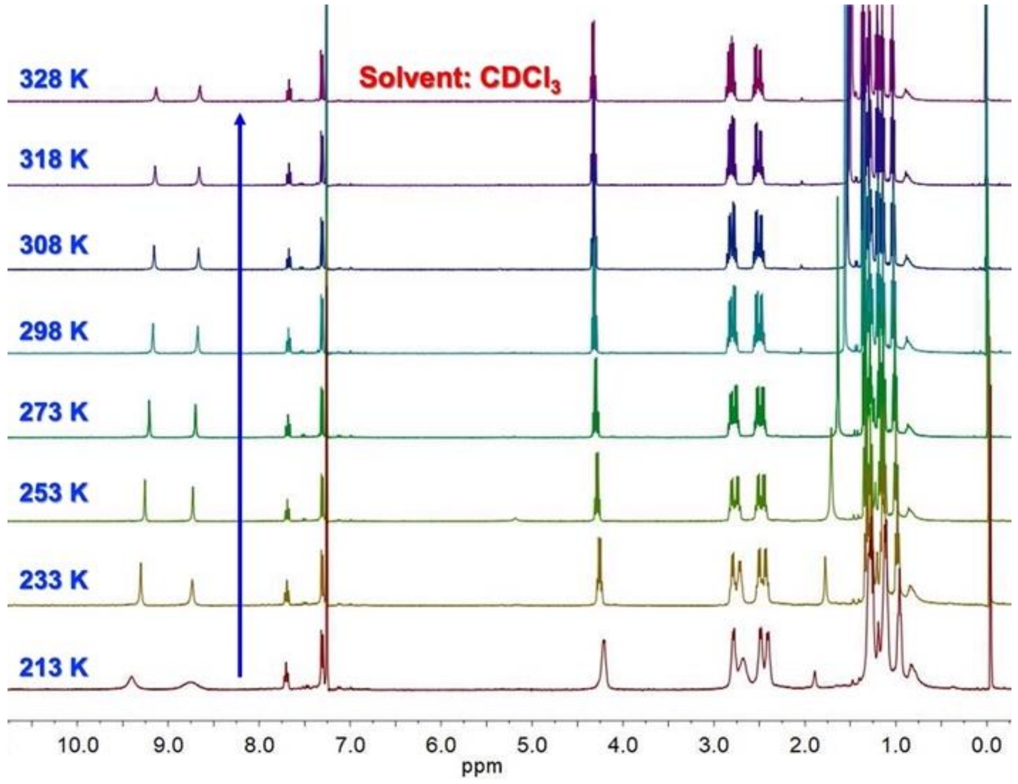

Figure S14. Variable temperature ^1H NMR (400 MHz in $CDCl_3$) spectra of (**10**)$_2$.

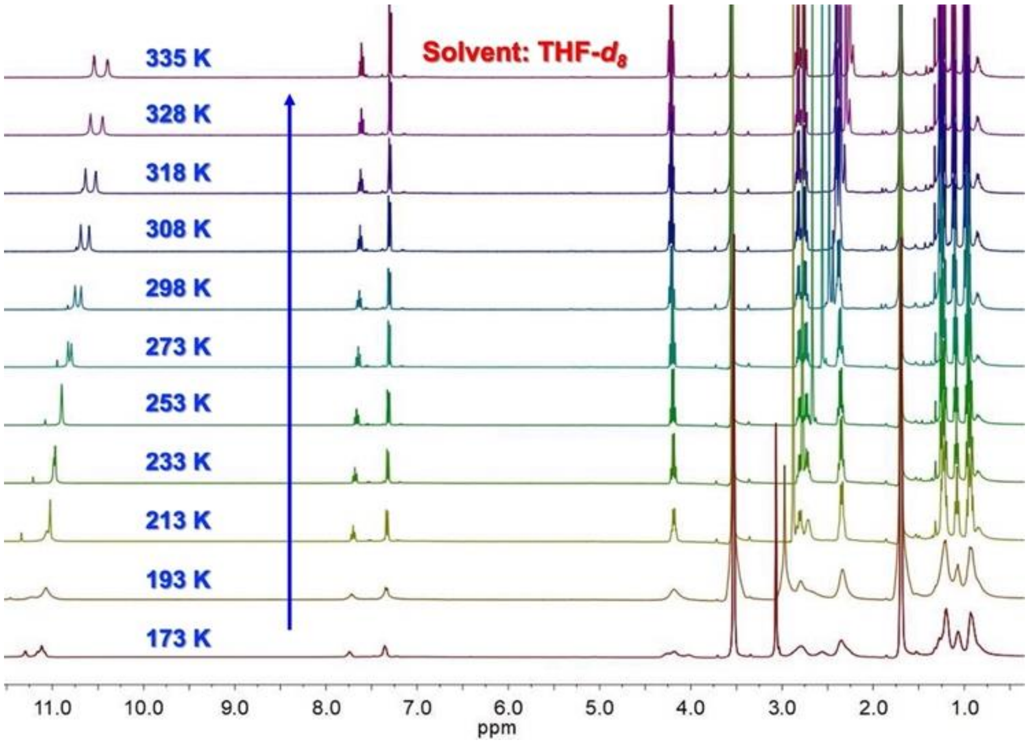

Figure S15. Variable temperature ^{1}H NMR (400 MHz in THF-d_8) spectra of (**10**)$_n$.

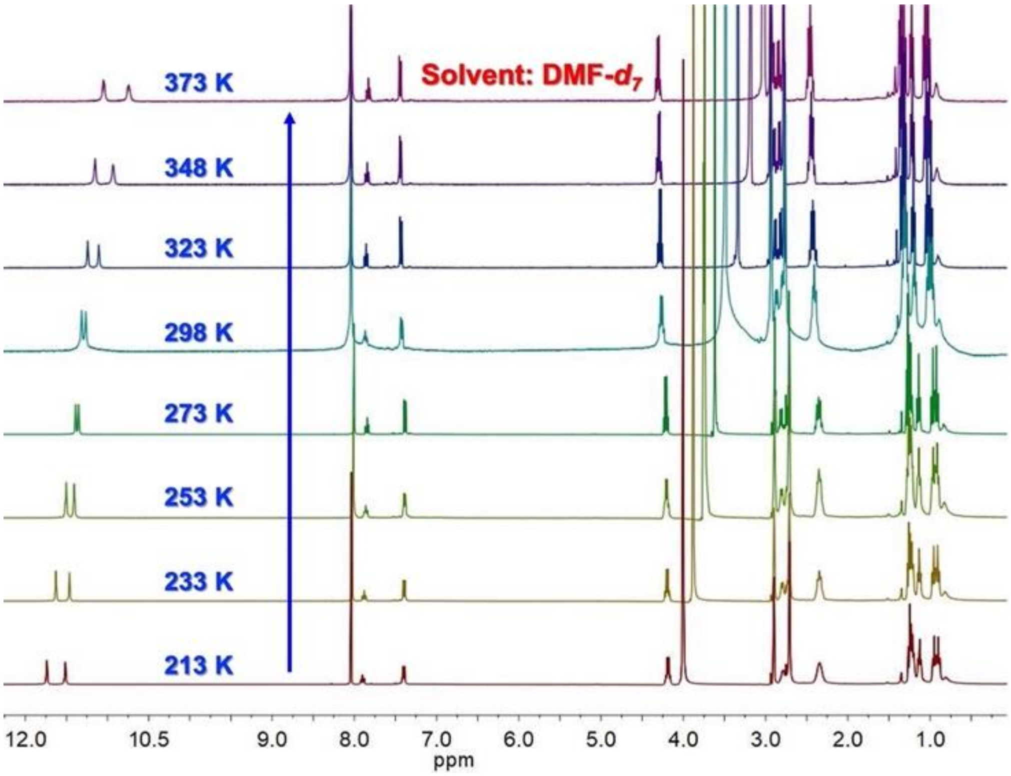

Figure S16. Variable temperature ^{1}H NMR (400 MHz in DMF-d_7) spectra of (**10**)$_n$.

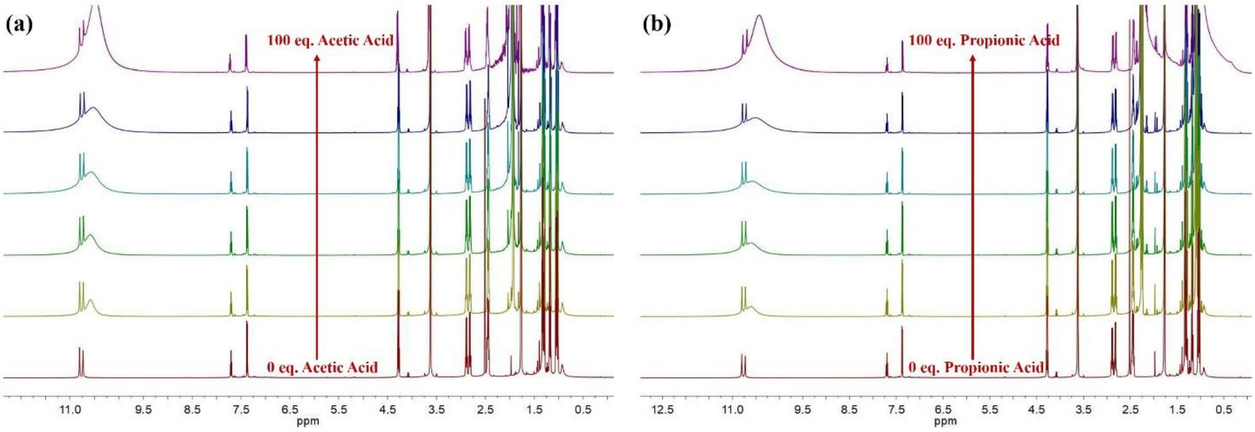

Figure S17. ^{1}H NMR (600 MHz, THF-d_8 at 298 K) spectroscopic titration results of $(\mathbf{10})_n$ against 100 equiv of acetic acid (a), and 100 equiv of propionic acid (b).

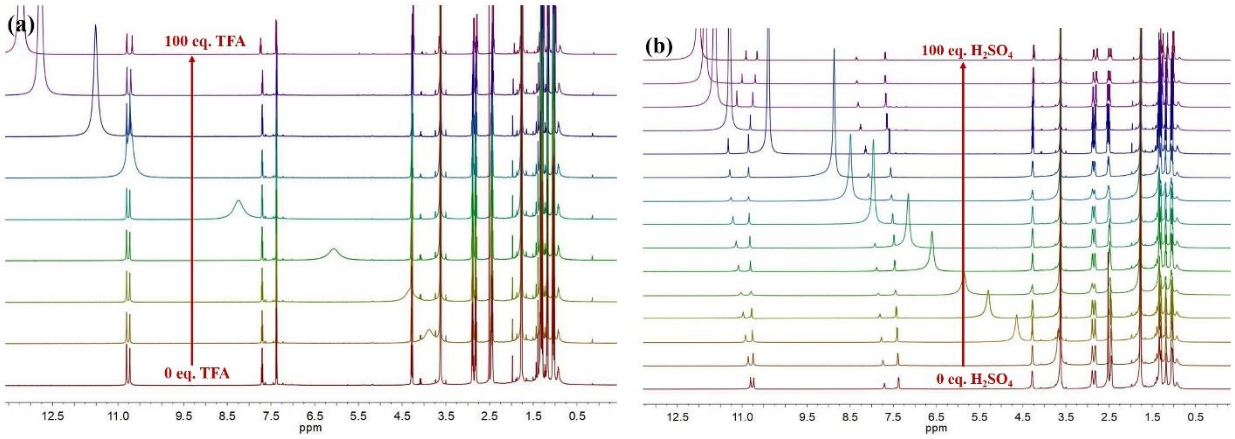

Figure S18. ^{1}H NMR (600 MHz, THF-d_8 at 298 K) spectroscopic titration results of **10** against 100 equiv of TFA (a), and 100 equiv of H_2SO_4 (b).

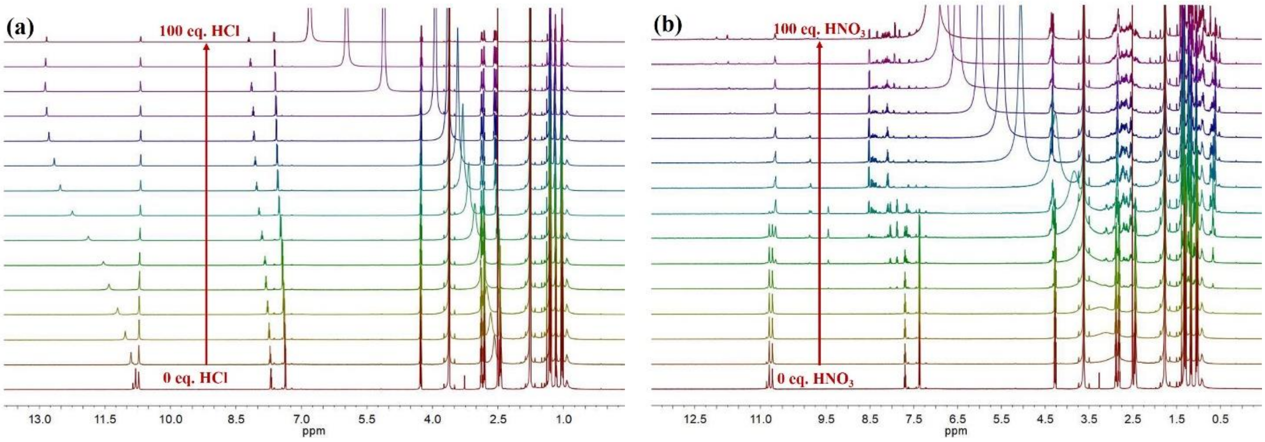

Figure S19. ^{1}H NMR (600 MHz, THF-d_8 at 298 K) spectroscopic titration results of **10** against 100 equiv of HCl (a), and 100 equiv of HNO_3 (b).

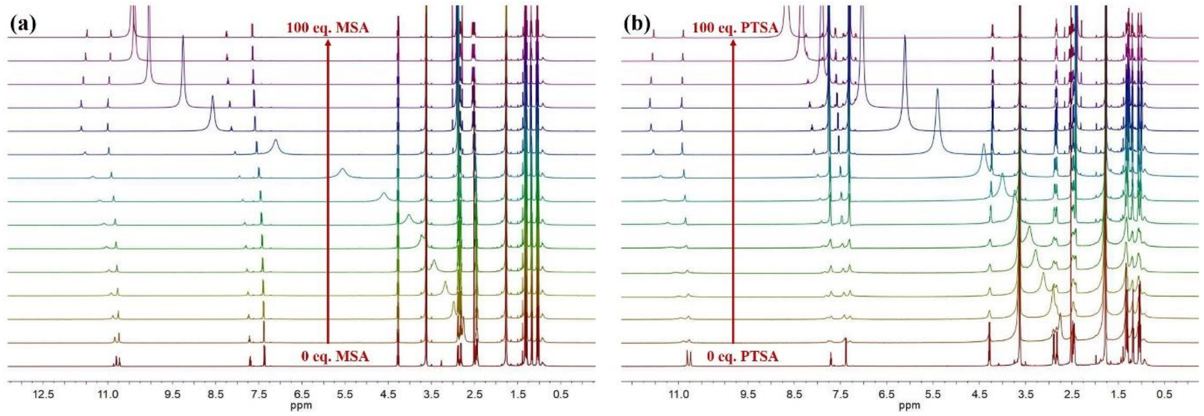

Figure S20. ^{1}H NMR (600 MHz, THF-d_8 at 298 K) spectroscopic titration results of **10** against 100 equiv of MSA (a), and 100 equiv of PTSA (b).

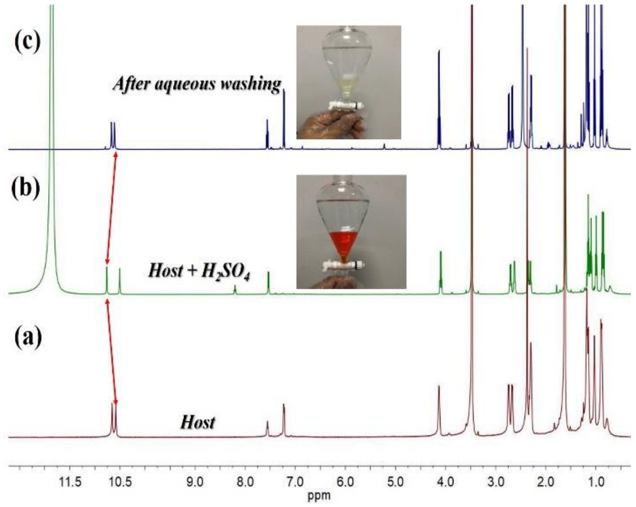

Figure S21. ^{1}H NMR (600 MHz, THF-d_8 at 298 K) spectra of **10** (a), with H_2SO_4 (b), and the organic phase after aqueous extraction (c). The spectrum (c) reveals that the supramolecular receptor releases H_2SO_4 upon aqueous wash.

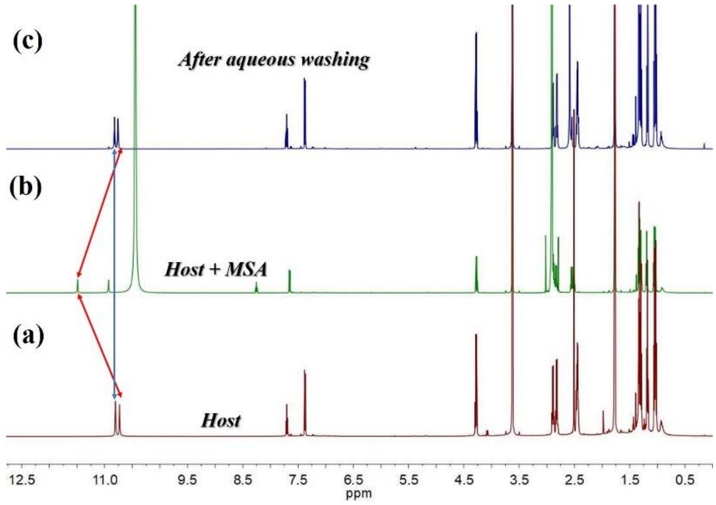

Figure S22. ^{1}H NMR (600 MHz, THF-d_8 at 298 K) spectra of **10** (a), with MSA (b), and the organic phase after aqueous extraction (c). The spectrum (c) reveals that the supramolecular receptor releases MSA upon aqueous wash.

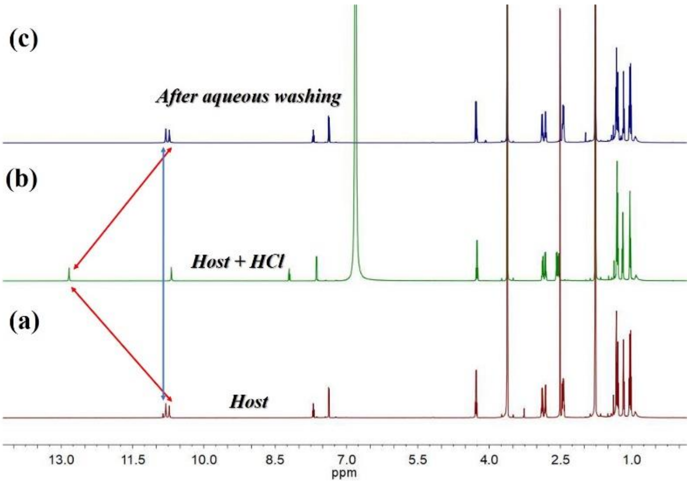

Figure S23. ^{1}H NMR (600 MHz, THF-d_8 at 298 K) spectra of **10** (a), with HCl (b), and the organic phase after aqueous extraction (c). The spectrum (c) reveals that the supramolecular receptor releases HCl upon aqueous wash.

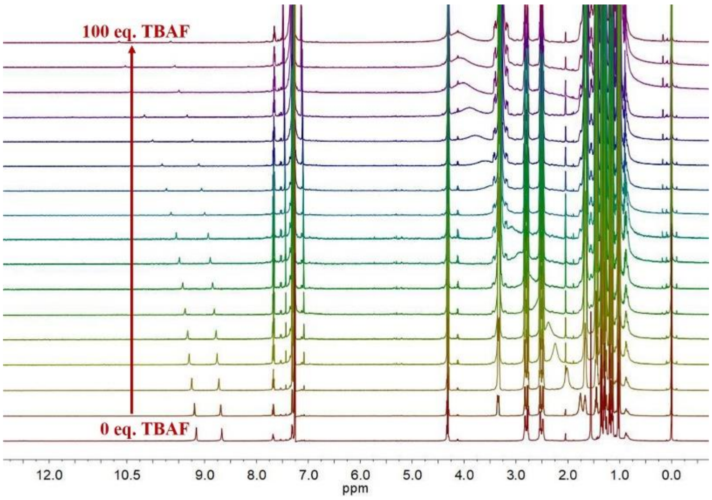

Figure S24. ^{1}H NMR (600 MHz, $CDCl_3$ at 298 K) spectroscopic titration result of **10** against 100 equiv of TBAF.

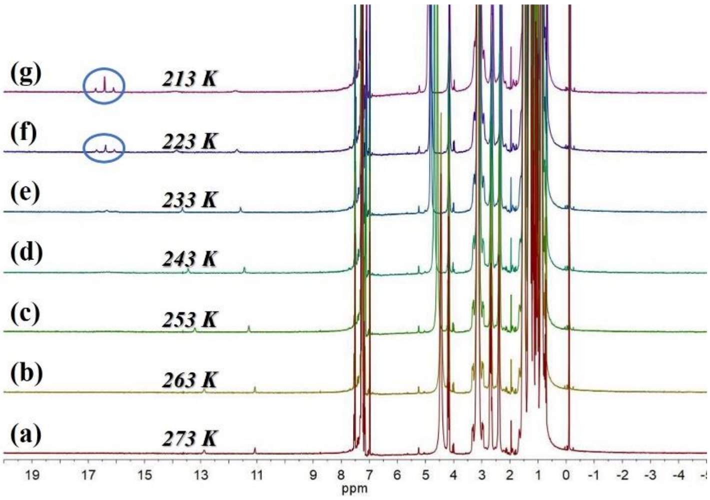

Figure S25. (a–g) Variable temperature ^{1}H NMR (400 MHz in $CDCl_3$) spectra of **10** with 100 equiv TBAF. The signal at 16.42 ppm corresponds to the generation of HF_2^-, which is visible at 233 K (f), and 213 K (g).

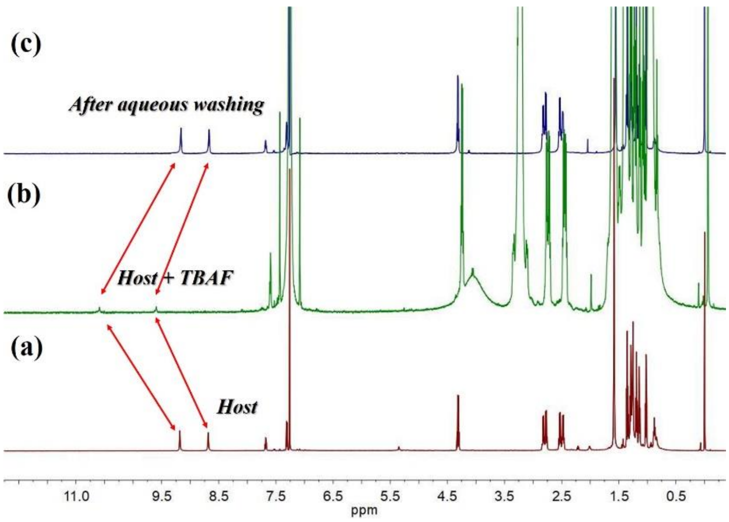

Figure S26. ^{1}H NMR (600 MHz, $CDCl_3$ at 298 K) spectra of **10** (a), with 100 equiv TBAF (b), and the organic phase after aqueous extraction (c). The spectrum (c) reveals that the supramolecular receptor releases TBAF upon aqueous wash.

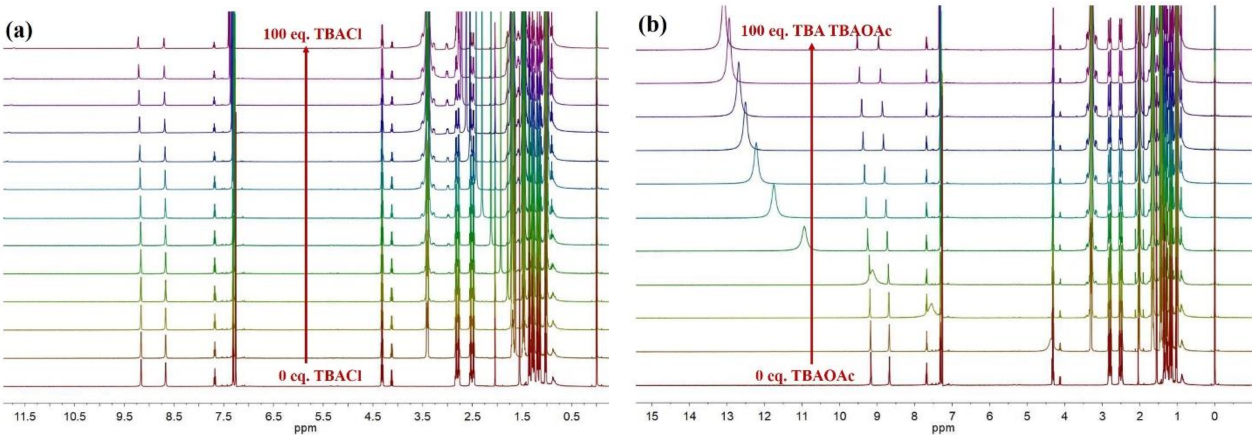

Figure S27. ^{1}H NMR (600 MHz, $CDCl_3$ at 298 K) spectroscopic titration results of **10** against 100 equiv of TBACl (a), and 100 equiv of TBAOAc (b).

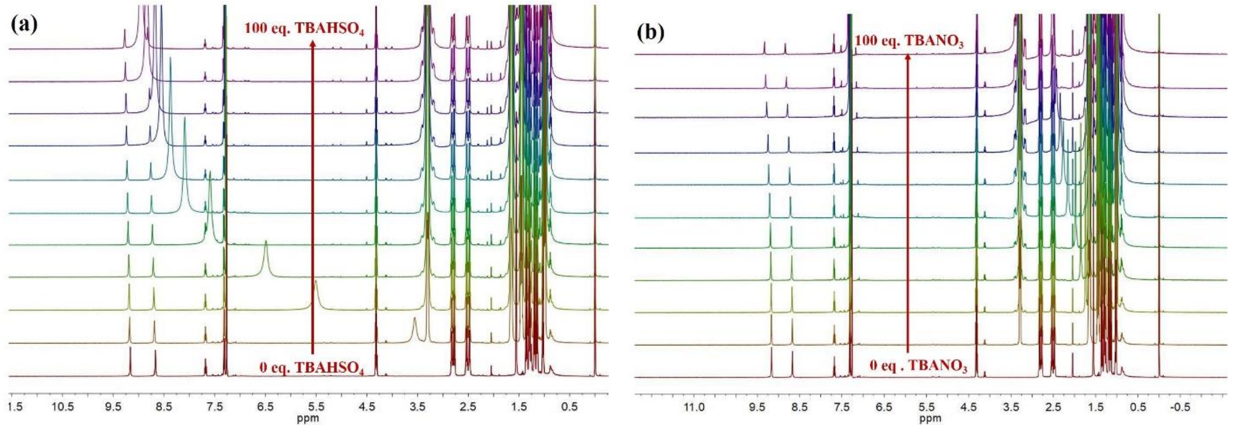

Figure S28. ^{1}H NMR (600 MHz, $CDCl_3$ at 298 K) spectroscopic titration results of **10** against 100 equiv of $TBAHSO_4$ (a), and 100 equiv of $TBANO_3$ (b).

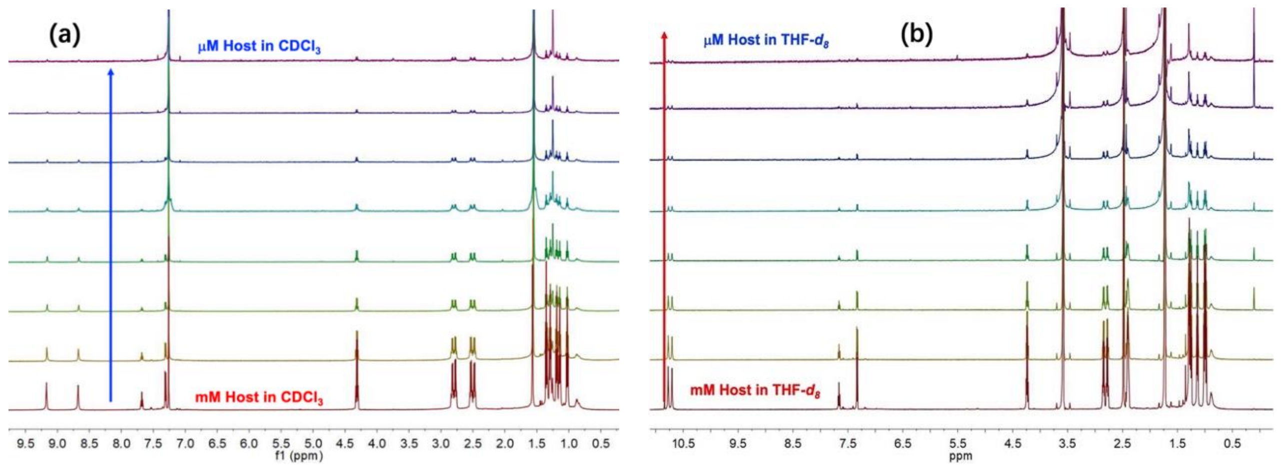

Figure S29. ^{1}H NMR spectra of **10** with different concetration from 5 mM to 5 μM performed in a) $CDCl_3$ and b) THF-d_8, via ^{1}H NMR spectroscopic method at 298 K.

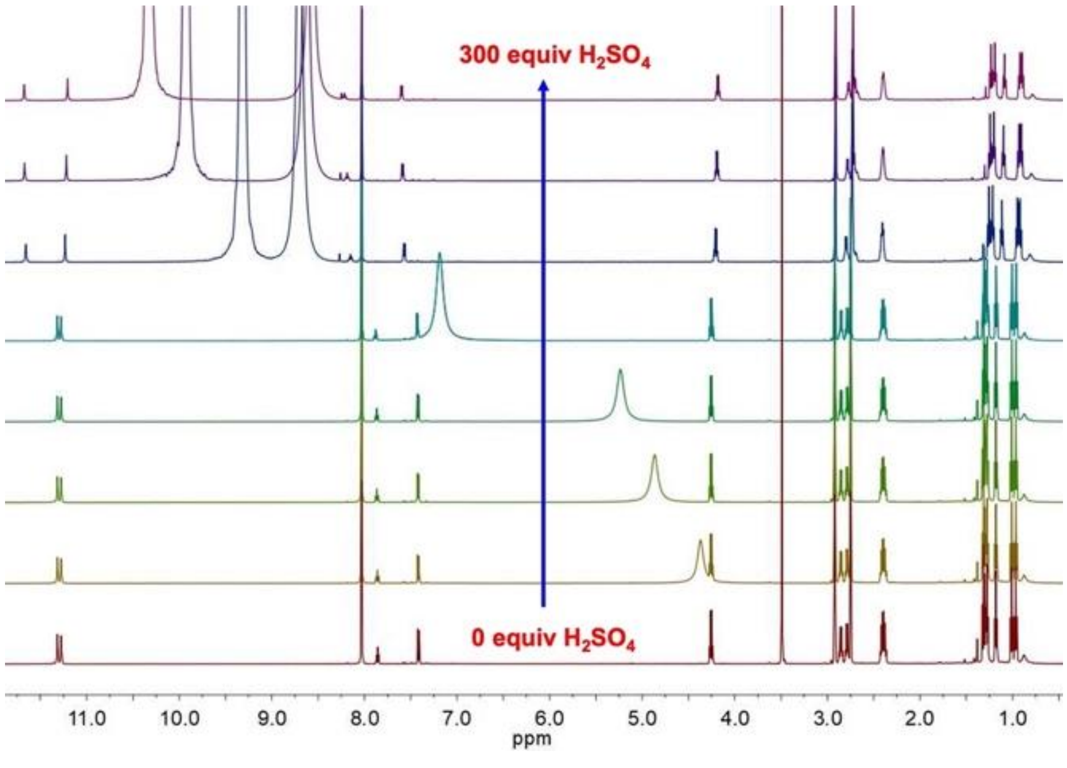

Figure S30. ^{1}H NMR (600 MHz, DMF-d_7 at 298 K) spectroscopic titration result of **10** against 300 equiv of H_2SO_4.

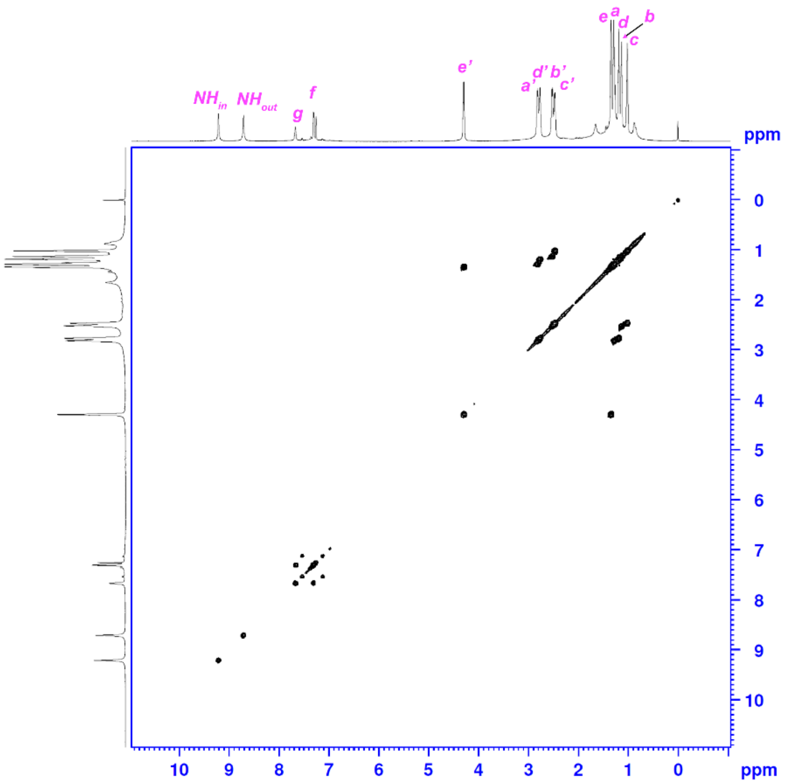

Figure S31. a) ^{1}H-^{1}H COSY (600 MHz, $CDCl_3$ at 298 K) spectra of receptor **10**.

II. Additional Photophysical Data from UV–vis Spectroscopic Analyses

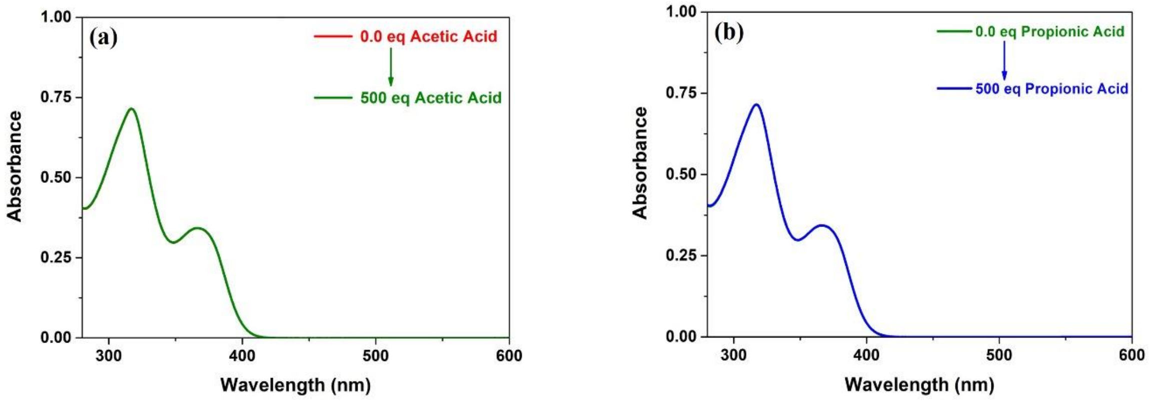

Figure S32. UV–vis spectroscopic titration (THF at 298 K) results of $(\mathbf{10})_n$ with acetic Acid (a), and propionic Acid (b).

III. Single Crystal X-ray Crystallography

(a) Single crystal X-ray diffraction analysis of the precursor 5:

Colorless rod-shaped crystals was obtained by vapor diffusion of hexanes into a THF solution of **5**. The data were collected on a Bruker D8 Venture diffractometer with a PHOTON 100 detector and a μ-focus Cu$K\alpha$ radiation source (λ = 1.5418 Å). The data were collected at 173 K. Crystallographic data are listed in the Table S1. Data were collected using Bruker APEX III software. Unit cell refinement and data reduction were performed using Bruker SAINT V8.37A software.[S1] The structure was solved by direct methods using SHELXT[S2] and refined by full-matrix least-squares on F^2 with anisotropic displacement parameters for the non-H atoms using SHELXL-2018/3. The hydrogen atoms were calculated in ideal positions with a riding mode.

Crystallographic data has been deposited in the Cambridge Crystallographic Data Center with CCDC number: 2056093. This data can be obtained free of charge at http://www.ccdc.cam.ac.uk/data_request/cif.

Table S1. Crystallographic data and structure refinement parameters for precursor **5**.

Identification code	Precursor **5**	
Empirical formula	$C_{27}H_{37}N_3O_5$	
Formula weight	483.59	
Temperature	173(2) K	
Wavelength	1.54184 Å	
Crystal system	Triclinic	
Space group	P-1	
Unit cell dimensions	a = 8.2281(7) Å	α = 104.145(14)°
	b = 15.7183(18) Å	β = 99.996(11)°
	c = 22.029(3) Å	γ = 102.736(8)°
Volume	2615.6(6) Å^3	
Z	4	
Calculated density	1.228 Mg/m^3	
Absorption coefficient	0.687 mm^{-1}	
F(000)	1040	
Crystal size	0.200 × 0.100 × 0.100 mm^3	
Theta range for data collection	3.016 to 68.416°	
Limiting indices	–9 ≤ h ≤ 9, –18 ≤ k ≤ 18, –26 ≤ l ≤ 26	
Reflections collected/unique	35010 / 9512 [R(int) = 0.0227]	
Completeness to θ = 67.679°	99.4%	
Absorption correction	Semi-empirical from equivalents	
Max. and min. transmission	0.935 and 0.875	
Refinement method	Full-matrix least-squares on F^2	

Data / restraints / parameters	9512 / 6 / 655
Goodness-of-fit on F^2	1.018
Final *R* indices [$I>2\sigma(I)$]	$R_1 = 0.0354$, $wR_2 = 0.1234$
R indices (all data)	$R_1 = 0.0384$, $wR_2 = 0.1289$
Extinction coefficient	n/a
Largest diff. peak and hole	0.213 and –0.175 e.Å^{-3}

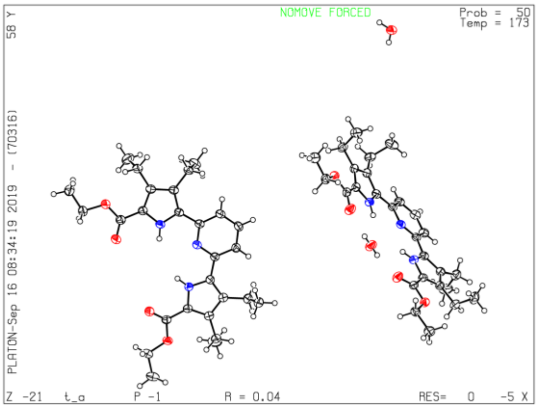

Figure S33. Asymmetric unit of **5** obtained from the single crystal X-ray diffraction analysis. The thermal ellipsoids are scaled to the 30% probability level.

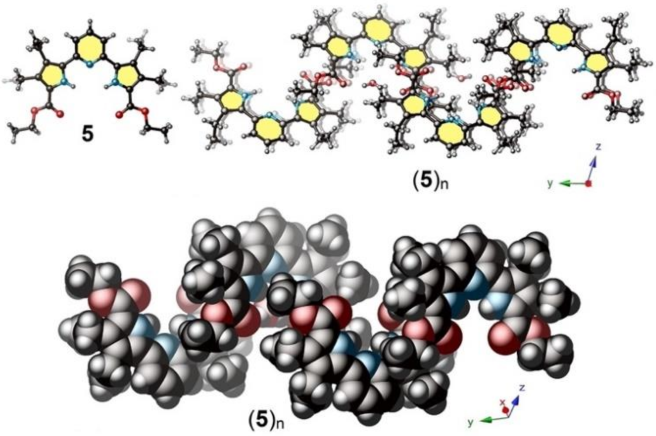

Figure S34. Different views of supramolecular network formed by precursor **5** obtained from a single crystal X-ray diffraction analysis.

(b) X-ray diffraction analysis of single crystals of 10 grown from $CHCl_3$: Yellow block shaped crystals was obtained by vapor diffusion of petroleum ether into an anhydrous $CHCl_3$ solution of **10**. The data were collected on a Rigaku XtaLAB AFC12 diffractometer with a μ-focus Cu$K\alpha$ radiation source (λ = 1.5418 Å) and a Kappa goniometer. The data were collected

at 296 K. Crystallographic data are listed in the Table S2. Data were collected using CrysAlisPro 1.171.39.32a software.[S3] Unit cell refinement and data reduction were performed using the same software. The structure was solved by direct methods using SHELXT[S2] and refined by full-matrix least-squares on F^2 with anisotropic displacement parameters for the non-H atoms using SHELXL-2018/3. The hydrogen atoms were calculated in ideal positions with a riding mode.

Crystallographic data has been deposited in the Cambridge Crystallographic Data Center with CCDC number: 2056094. This data can be obtained free of charge at http://www.ccdc.cam.ac.uk/data_request/cif.

Table S2. Crystallographic data and structure refinement parameters for receptor **10** grown from anhydrous $CHCl_3$.

Identification code	**10** in $CHCl_3$	
Empirical formula	$C_{43}H_{47}N_5O_4$	
Formula weight	697.85	
Temperature	296(2) K	
Wavelength	1.54184 Å	
Crystal system	Monoclinic	
Space group	C2/m	
Unit cell dimensions	a = 14.0979(9) Å	α = 90°
	b = 24.7324(12) Å	β = 100.784 (6)°
	c = 15.4405(8) Å	γ = 90°
Volume	5288.6(5) $Å^3$	
Z	4	
Calculated density	0.876 g/m^3	
Absorption coefficient	0.452 mm^{-1}	
F(000)	1488	
Crystal size	0.350 × 0.240 × 0.150 mm^3	
Theta range for data collection	3.658 to 66.600°	
Limiting indices	$-16 \leq h \leq 16, -29 \leq k \leq 26, -18 \leq l \leq 14$	
Reflections collected/unique	26035 / 4780 [R(int) = 0.0581]	
Completeness to θ = 66.600°	99.9%	
Absorption correction	Semi-empirical from equivalents	
Max. and min. transmission	0.935 and 0.858	
Refinement method	Full-matrix least-squares on F^2	
Data/restraints/parameters	4780 / 0 / 242	
Goodness-of-fit on F^2	1.021	
Final R indices [$I>2\sigma(I)$]	$R_1 = 0.0931$, $wR_2 = 0.2403$	

R indices (all data)	R_1 = 0.1162, wR_2 = 0.2623
Extinction coefficient	n/a
Largest diff. peak and hole	0.593 and –0.232 e.Å^{-3}

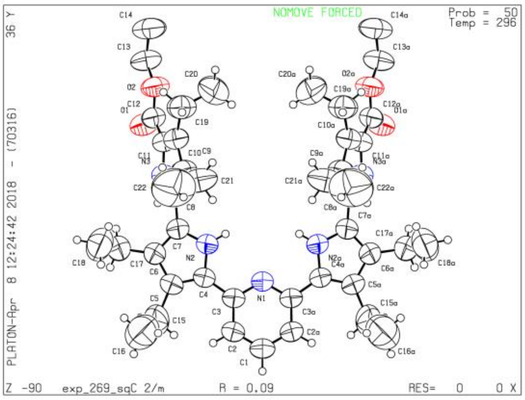

Figure S35. Asymmetric unit of **10** obtained from the single crystal X-ray diffraction analysis. The thermal ellipsoids are scaled to the 30% probability level.

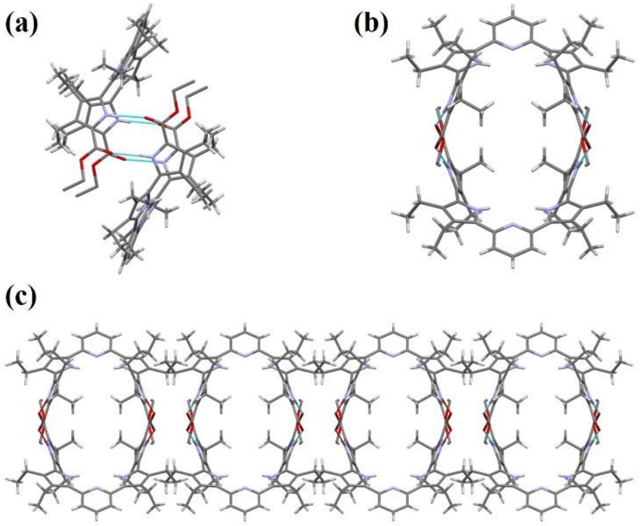

Figure S36. Different views of the single crystal X-ray structure of (**10**)$_2$ dimeric unit (a, b); crystal-packing diagram (viewed along the a-axis) of (**10**)$_2$ grown from anhydrous $CHCl_3$ solvent (c).

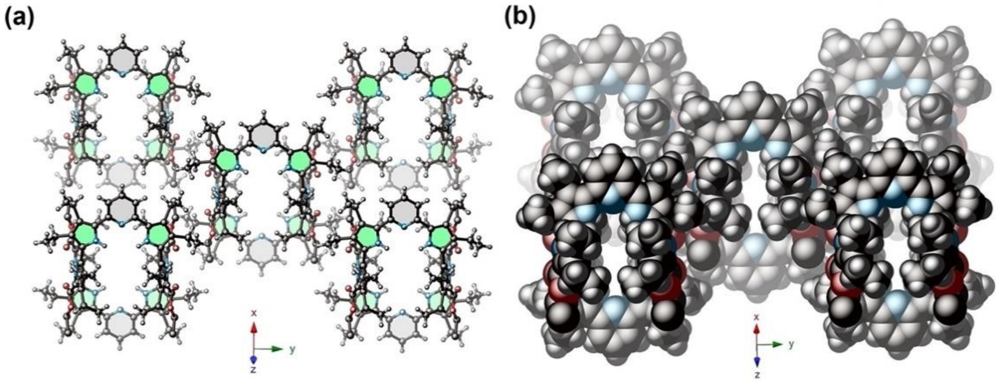

Figure 37. (a,b) Ball-and-stick and space-filling model representing of the crystal packing of the discrete pseudo-macrocyclic cage-like structure of hydrogen-bonded supramolecular dimeric units of (**10**)$_2$ grown from $CHCl_3$. Solvent molecules ($CHCl_3$) are omitted for clarity.

(c) Single crystal X-ray diffraction analysis of (10)$_n$ grown from anhydrous DMF:

Light-yellow block shaped crystals was obtained by slow evaporation of DMF solution of receptor **10**. The data were collected on a Bruker D8 Venture diffractometer with a μ-focus Cu$K\alpha$ radiation source (λ = 1.5418 Å). The data were collected at 150 K. Crystallographic data are listed in the Table S3. Data were collected using APEX-II CCD software. Unit cell refinement and data reduction were performed using SAINT V8.40B software. The structure was solved by direct methods using SHELXT[S2] and refined by full-matrix least-squares on F^2 with anisotropic displacement parameters for the non-H atoms using SHELXL-2018/2. The hydrogen atoms were calculated in ideal positions with a riding mode.

Crystallographic data has been deposited in the Cambridge Crystallographic Data Center with CCDC number: 2058193. This data can be obtained free of charge at http://www.ccdc.cam.ac.uk/data_request/cif.

Table S3. Crystal data and structure refinement for (**10**)$_n$ grown from anhydrous DMF.

Identification code	**10** in DMF	
Empirical formula	$C_{95}H_{135}N_{13}O_{11}$	
Formula weight	1635.15	
Temperature	150 K	
Wavelength	1.54178 Å	
Crystal system	Triclinic	
Space group	P-1	
Unit cell dimensions	a = 14.4978(6) Å	α = 107.090(2)°
	b = 15.2219(6) Å	β = 104.589(2)°
	c = 23.1319(9) Å	γ = 93.241(2)°
Volume	4674.8(3) Å^3	
Z	2	
Calculated density	1.162 g/m^3	
Absorption coefficient	0.608 mm^{-1}	
F(000)	1768	
Crystal size	0.12 × 0.07 × 0.06 mm^3	
Theta range for data collection	4.164 to 133.632°	
Limiting indices	$-17 \leq h \leq 17, -18 \leq k \leq 18, -27 \leq l \leq 27$	

Reflections collected/unique	52341 / 16478 [R(int) = 0.0462]
Completeness to θ = 66.816°	99.2%
Absorption correction	Semi-empirical from equivalents
Max. and min. transmission	0.664 and 0.753
Refinement method	Full-matrix least-squares on F^2
Data/restraints/parameters	16478 / 284 / 1223
Goodness-of-fit on F^2	1.035
Final R indices [$I>2\sigma(I)$]	R_1 = 0.0621, wR_2 = 0.1787
R indices (all data)	R_1 = 0.0720, wR_2 = 0.1858
Extinction coefficient	n/a
Largest diff. peak and hole	0.75 and –0.29 e.Å^{-3}

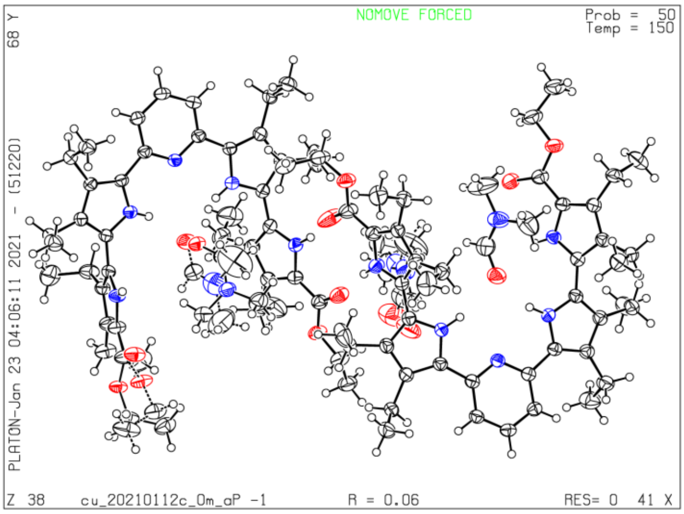

Figure S38. Asymmetric unit of (**10**)$_n$ obtained from the single crystal X-ray diffraction analysis. The thermal ellipsoids are scaled to the 30% probability level.

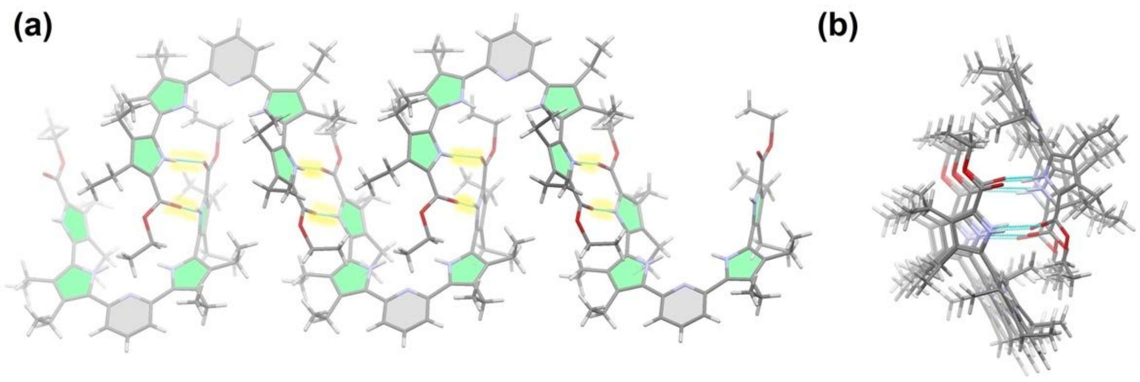

Figure S39. Two different views of hydrogen-bonded 1D infinite polymeric chain of (**10**)$_n$ grown from DMF. For clarification the solvent molecules are omitted.

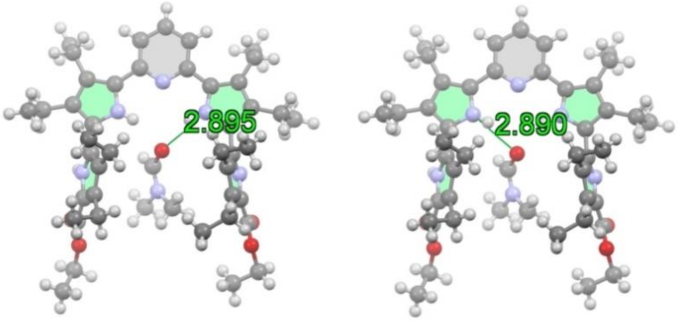

Figure S40. The hydrogen-bonding and distance between "O" (of DMF guest) and "N" (of pyrrole) determined from the single crystal X-ray structure of $(\mathbf{10})_n$ grown from anhydrous DMF.

IV. Additional Data from DFT Calculations

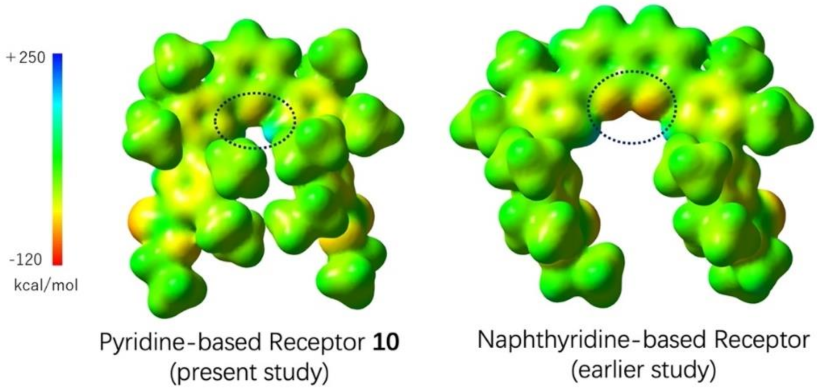

Figure S41. Electrostatic potential maps of pyridine-based receptor **10** (left) used under present studies and naphthyridine-based receptor **11** (right) reported earlier.

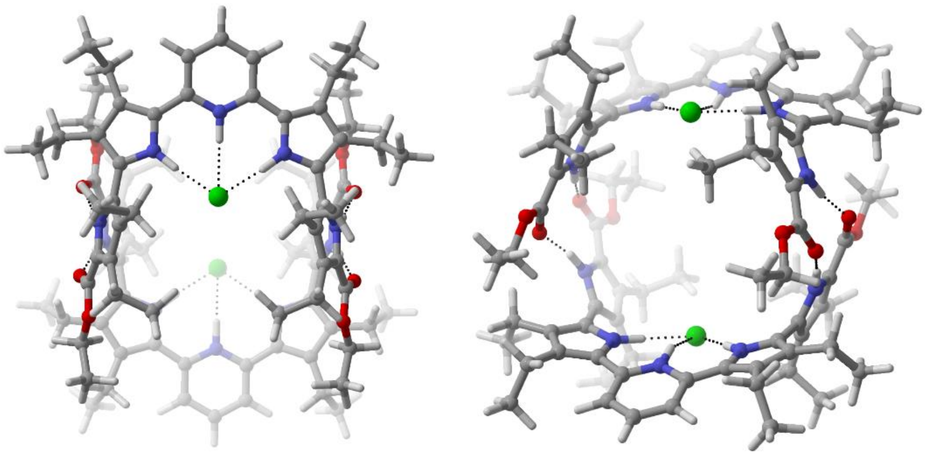

Figure S42. Different views of theoretically predicted energy minimized supramolecular structures of receptor–acid complex with the chemical formula $[(\mathbf{10}{\bullet}H)^{+}(Cl^{-})]_2$

V. References & Notes

S1. Bruker SADABS, SMART and SAINT. Bruker AXS Inc., Madison, Wisconsin, USA, **2017**.

S2. Sheldrick, G. M., SHELXT-integrated space-group and crystal-structure determination. *Acta Cryst. A*, **2015**, *71*, 3–8.

S3. CrysAlisPro. Agilent Technologies (2013). Agilent Technologies UK Ltd., Oxford, UK, SuperNova CCD System, CrysAlicPro Software System, 1.171.39.46.

V. Cartesian Coordinate for 10

Symbol	X	Y	Z
O	-4.84804	3.725273	-1.899
O	-3.90385	4.962539	-0.2659
N	-0.28248	-3.49667	-0.60509
N	-2.28307	-1.76231	-0.28834
H	-1.33602	-1.47855	-0.08208
N	-3.89545	1.408963	-0.71907
H	-4.34807	1.282671	-1.6146
C	-0.59823	-6.22942	-0.80508
H	-0.72253	-7.30576	-0.87764
C	-1.7151	-5.4034	-0.80132
H	-2.71407	-5.81895	-0.83641
C	-1.51193	-4.02006	-0.71159
C	-2.59513	-3.03523	-0.67892
C	-3.95375	-3.05377	-0.98493
C	-4.45466	-1.74106	-0.75336
C	-3.3841	-0.96244	-0.32634
C	-3.31041	0.445588	0.04185
C	-2.71909	1.071612	1.139889
C	-2.97294	2.463521	1.021524
C	-3.69511	2.639629	-0.15255
C	-4.21434	3.800383	-0.8659
C	-4.37748	6.141453	-0.92974
C	-3.91196	7.326087	-0.11105
C	-4.77127	-4.22292	-1.45445
H	-5.5571	-3.86338	-2.12797

H	-4.1519	-4.89496	-2.05783
C	-5.41608	-4.99183	-0.2953
H	-4.65471	-5.36847	0.395742
H	-6.00688	-5.83927	-0.65616
H	-6.07619	-4.33243	0.277667
C	-5.88721	-1.28878	-0.84173
H	-5.99591	-0.45671	-1.54857
H	-6.49613	-2.1029	-1.24965
C	-6.44956	-0.86872	0.521453
H	-6.38376	-1.69837	1.232468
H	-7.49732	-0.56468	0.439804
H	-5.88125	-0.02888	0.934485
C	-2.51274	3.529912	1.973966
H	-3.26634	4.319112	2.034529
H	-2.41764	3.099166	2.977325
C	-1.17362	4.139238	1.544455
H	-0.39572	3.36894	1.490198
H	-0.84306	4.909334	2.24851
H	-1.26775	4.592134	0.552993
C	-2.07975	0.38989	2.317138
H	-1.49719	-0.4806	1.99527
H	-1.36268	1.074609	2.786083
C	-3.12256	-0.06009	3.346444
H	-3.70445	0.795898	3.702913
H	-2.65136	-0.54227	4.208289
H	-3.81897	-0.77079	2.889763
O	6.37892	2.169276	-1.65356
O	5.214814	3.905302	-0.80441
N	2.014123	-2.21886	-0.45536
H	1.169618	-1.72019	-0.69463
N	4.474692	0.401386	-0.6986
H	5.20331	-0.01072	-1.26577
C	0.676219	-5.6873	-0.70038
H	1.551115	-6.32438	-0.69881
C	0.795681	-4.29584	-0.58784
C	2.065682	-3.58355	-0.43332
C	3.391731	-3.94229	-0.18939
C	4.13389	-2.73404	-0.04936

C	3.240471	-1.68042	-0.2249
C	3.407741	-0.23623	-0.14701
C	2.603358	0.729332	0.461956
C	3.224816	1.989141	0.257504
C	4.380626	1.749935	-0.47506
C	5.425478	2.598023	-1.03556
C	6.205436	4.790904	-1.34203
C	5.778268	6.198786	-0.98743
C	3.93602	-5.33126	-0.00665
H	5.024278	-5.30866	-0.1218
H	3.575778	-5.99044	-0.80439
C	3.587207	-5.92063	1.364981
H	2.502707	-5.96152	1.508743
H	3.989069	-6.9321	1.478567
H	4.001135	-5.29401	2.160911
C	5.576674	-2.58577	0.351236
H	6.121579	-1.96449	-0.37015
H	6.066936	-3.56402	0.321401
C	5.729479	-1.98367	1.753164
H	5.224447	-2.6116	2.493655
H	6.783405	-1.8967	2.033602
H	5.28022	-0.98623	1.797735
C	2.689706	3.317854	0.710145
H	3.514176	3.958872	1.033775
H	2.040749	3.16537	1.580582
C	1.900431	4.024851	-0.39737
H	1.067322	3.402136	-0.73976
H	1.491514	4.975917	-0.04133
H	2.552083	4.224367	-1.25289
C	1.386917	0.486865	1.313105
H	0.911182	-0.46173	1.042245
H	0.638343	1.268088	1.124961
C	1.737263	0.438799	2.80491
H	2.197327	1.377542	3.129134
H	0.848094	0.259422	3.417707
H	2.454389	-0.36619	2.993597
H	7.179603	4.534307	-0.91519
H	6.267098	4.637846	-2.42353

H	4.796609	6.42048	-1.41435
H	5.718992	6.320505	0.097228
H	6.499759	6.920327	-1.37965
H	-3.9764	6.159303	-1.94741
H	-5.46793	6.092775	-1.00427
H	-4.3229	7.279328	0.90077
H	-2.82109	7.33819	-0.04099
H	-4.24152	8.258128	-0.5776

Cartesian Coordinate for 11

Symbol	X	Y	Z
O	-7.08367	2.025396	-1.7324
O	-6.28293	3.856709	-0.67791
O	2.469746	3.788331	-2.31091
O	3.292948	5.428342	-0.9921
N	-5.30463	0.424521	-0.35569
H	-5.86422	-0.05008	-1.05146
N	-2.79026	-2.04801	0.26834
H	-1.93164	-1.52233	0.164287
N	-0.45947	-3.30117	0.079407
N	1.810888	-2.96925	-0.07522
N	3.697121	-1.10312	-0.09912
H	2.728497	-0.8532	0.056432
N	3.847842	1.889862	-0.8513
H	3.356256	1.539031	-1.66263
C	-5.36627	1.775269	-0.13797
C	-4.40731	2.106906	0.814856
C	-3.74884	0.896845	1.168608
C	-4.32983	-0.12657	0.41723
C	-4.05556	-1.55851	0.368846
C	-4.92821	-2.64346	0.41428
C	-4.13825	-3.82462	0.329359
C	-2.8045	-3.41699	0.243095
C	-1.51513	-4.09901	0.153183
C	-1.4008	-5.52277	0.154961

H	-2.28319	-6.144	0.222577
C	-0.15608	-6.08342	0.074314
H	-0.02981	-7.16326	0.075409
C	0.984308	-5.24995	-0.00954
C	2.31544	-5.722	-0.09207
H	2.50367	-6.79277	-0.09138
C	3.34808	-4.82816	-0.16682
H	4.372616	-5.16973	-0.22176
C	3.049647	-3.43172	-0.15993
C	0.778694	-3.84245	-0.00211
C	4.092274	-2.40831	-0.22083
C	5.483916	-2.41613	-0.34562
C	5.913906	-1.05953	-0.28404
C	4.777827	-0.27371	-0.12219
C	4.635085	1.175484	0.000561
C	5.193068	2.06429	0.91782
C	4.725514	3.366101	0.584731
C	3.894706	3.219027	-0.52183
C	-6.32618	2.53705	-0.92786
C	-7.18601	4.676014	-1.43967
H	-8.15509	4.174515	-1.49527
H	-7.28115	5.590431	-0.85116
C	-6.63157	4.958387	-2.82387
C	3.146093	4.145337	-1.36392
C	2.555918	6.39744	-1.75703
H	3.077446	7.338459	-1.57398
H	2.627328	6.139638	-2.81622
C	1.10981	6.462377	-1.30078
H	0.606466	5.512512	-1.49306
H	0.583284	7.249907	-1.84742
H	1.055588	6.687977	-0.23235
C	-4.1341	3.467354	1.391928
H	-3.16629	3.440577	1.905129
H	-4.04566	4.197067	0.581022
C	-5.22429	3.934523	2.365511
H	-6.17761	4.038849	1.841752
H	-4.96462	4.902715	2.804189
H	-5.36112	3.214311	3.177458

C	-2.67805	0.73524	2.211765
H	-2.24577	-0.26711	2.136058
H	-1.86021	1.440033	2.016849
C	-3.19845	0.938366	3.639916
H	-3.57985	1.953602	3.780737
H	-2.40332	0.771265	4.372393
H	-4.0146	0.24099	3.850371
C	-6.4195	-2.56473	0.579897
H	-6.73684	-3.26378	1.363389
H	-6.68153	-1.56482	0.94328
C	-7.20109	-2.8663	-0.70469
H	-6.90781	-2.19078	-1.51512
H	-8.27778	-2.75838	-0.54549
H	-7.00942	-3.8869	-1.04933
C	-4.69868	-5.21982	0.308255
H	-4.16018	-5.86413	1.012515
H	-5.7299	-5.18348	0.676724
C	-4.69368	-5.84649	-1.09347
H	-5.28198	-5.23753	-1.78555
H	-5.11708	-6.85535	-1.07662
H	-3.67975	-5.90453	-1.50039
C	5.044762	4.638499	1.317833
H	5.321918	5.415444	0.598684
H	5.91989	4.463701	1.953914
C	3.875334	5.144736	2.172596
H	3.022936	5.392102	1.53487
H	4.16043	6.042268	2.729741
H	3.552704	4.382824	2.888226
C	6.071292	1.687601	2.077514
H	6.996018	2.278702	2.052731
H	6.369228	0.639819	1.96529
C	5.385135	1.866747	3.436888
H	5.126403	2.914023	3.616474
H	6.038282	1.536537	4.249975
H	4.461752	1.281433	3.47729
C	7.317258	-0.54418	-0.4362
H	7.985023	-1.05972	0.266212
H	7.333296	0.514366	-0.15638

C	7.86269	-0.68634	-1.86239
H	7.922627	-1.73606	-2.16377
H	8.865084	-0.25546	-1.94205
H	7.208731	-0.17133	-2.57225
C	6.399517	-3.58947	-0.56444
H	7.429838	-3.25704	-0.39676
H	6.219126	-4.36521	0.188938
C	6.289153	-4.18762	-1.97393
H	5.274366	-4.54186	-2.17797
H	6.978849	-5.02753	-2.10116
H	6.52287	-3.43208	-2.72909
H	-5.64718	5.428144	-2.75324
H	-6.54671	4.032416	-3.39633
H	-7.3017	5.636757	-3.35966

Cartesian Coordinate for [10•HCl]$_2$

Symbol	X	Y	Z
O	-1.15441	5.245714	-1.29865
O	-1.41165	4.797068	-3.48451
N	5.410176	-0.00458	0.300831
N	4.290339	2.433143	-0.71263
H	3.961849	1.635658	-1.29396
N	1.53025	4.457005	-1.45885
H	1.261448	4.738148	-0.51497
C	7.751151	-0.00661	1.66857
H	8.706317	-0.00744	2.184355
C	7.163533	1.203651	1.328471
H	7.655445	2.138671	1.548858
C	5.953483	1.210862	0.62673
C	5.269453	2.42168	0.248671
C	5.371944	3.733288	0.760033
C	4.436257	4.526099	0.058597
C	3.785808	3.679914	-0.84467
C	2.72575	3.926616	-1.82076
C	2.686506	3.611802	-3.17945
C	1.395529	3.958233	-3.64535

C	0.699557	4.470724	-2.54948
C	-0.67918	4.878114	-2.36606
C	-2.80912	5.096757	-3.3502
C	-3.44544	4.821304	-4.69588
C	6.258705	4.252676	1.856254
H	5.712963	5.031832	2.40059
H	6.461247	3.468187	2.59294
C	7.566999	4.855102	1.326266
H	8.13993	4.124086	0.746546
H	8.195968	5.220938	2.143113
H	7.34944	5.693358	0.657422
C	4.204909	6.006835	0.182516
H	3.158783	6.196074	0.4471
H	4.808627	6.398952	1.009583
C	4.559427	6.761219	-1.10563
H	5.604998	6.590622	-1.38195
H	4.404325	7.83692	-0.98257
H	3.933184	6.420842	-1.93627
C	0.879935	3.700512	-5.03181
H	0.198352	4.498314	-5.33786
H	1.725059	3.710155	-5.72888
C	0.165494	2.344465	-5.11571
H	0.831308	1.538774	-4.79177
H	-0.16483	2.136862	-6.13839
H	-0.7105	2.33879	-4.46052
C	3.819618	3.091214	-4.01598
H	4.524435	2.537668	-3.38962
H	3.433791	2.358963	-4.73397
C	4.556484	4.220909	-4.74691
H	3.87224	4.790743	-5.38397
H	5.361432	3.826369	-5.37447
H	4.993897	4.918503	-4.02516
O	-1.16358	-5.24254	-1.29928
O	-1.4199	-4.79346	-3.48513
N	4.286247	-2.44023	-0.71288
H	3.959112	-1.64211	-1.29412
N	1.522626	-4.45907	-1.45941
H	1.25331	-4.73986	-0.51557

C	7.161454	-1.21585	1.32843
H	7.651753	-2.15172	1.548808
C	5.951426	-1.22096	0.626641
C	5.265363	-2.43057	0.248424
C	5.365588	-3.74242	0.759619
C	4.428601	-4.53355	0.058016
C	3.779595	-3.68612	-0.84513
C	2.719094	-3.93079	-1.82126
C	2.680404	-3.61568	-3.17989
C	1.388759	-3.95959	-3.64581
C	0.691878	-4.47101	-2.55003
C	-0.68761	-4.87587	-2.36667
C	-2.81795	-5.09042	-3.35082
C	-3.45376	-4.81363	-4.69646
C	6.251464	-4.26347	1.855767
H	5.704443	-5.04184	2.399948
H	6.455271	-3.47946	2.592615
C	7.558785	-4.86792	1.325687
H	8.132907	-4.13773	0.746107
H	8.18715	-5.23493	2.142473
H	7.339871	-5.7057	0.656685
C	4.194803	-6.01392	0.181629
H	3.148387	-6.20147	0.446241
H	4.797937	-6.40723	1.008558
C	4.547978	-6.76861	-1.10671
H	5.593814	-6.5997	-1.38306
H	4.391088	-7.84407	-0.98387
H	3.922254	-6.42701	-1.93724
C	0.873509	-3.70043	-5.03213
H	0.190704	-4.49704	-5.33856
H	1.718593	-3.71099	-5.72923
C	0.161119	-2.34325	-5.11534
H	0.828139	-1.53875	-4.79093
H	-0.16884	-2.13461	-6.13792
H	-0.71491	-2.33661	-4.46019
C	3.814427	-3.09696	-4.01634
H	4.520735	-2.54567	-3.38967
H	3.430097	-2.36312	-4.73351

C	4.548461	-4.22769	-4.74852
H	3.862715	-4.7954	-5.38586
H	5.354097	-3.83443	-5.37599
H	4.984513	-4.92692	-4.02753
O	1.16373	5.242272	1.299372
O	1.419821	4.793188	3.485243
N	-5.41022	0.004401	-0.30097
N	-4.28623	2.439998	0.71269
H	-3.95899	1.641846	1.293829
N	-1.52257	4.458791	1.459295
H	-1.25332	4.739851	0.515522
C	-7.75122	0.006413	-1.66866
H	-8.70639	0.007232	-2.18443
C	-7.16155	1.21566	-1.3285
H	-7.65188	2.151523	-1.54882
C	-5.95148	1.22078	-0.62677
C	-5.26541	2.43039	-0.24855
C	-5.3657	3.742271	-0.75965
C	-4.42866	4.533364	-0.05807
C	-3.77958	3.685883	0.844966
C	-2.71905	3.930507	1.821086
C	-2.68036	3.615233	3.179674
C	-1.38873	3.959112	3.645648
C	-0.69184	4.47067	2.549933
C	0.687653	4.875574	2.366699
C	2.817927	5.089994	3.351053
C	3.453608	4.81286	4.696686
C	-6.25168	4.263382	-1.85569
H	-5.70476	5.041874	-2.39979
H	-6.45544	3.479451	-2.59263
C	-7.55903	4.867645	-1.32546
H	-8.13304	4.137332	-0.74592
H	-8.18748	5.234685	-2.14216
H	-7.34015	5.705368	-0.65637
C	-4.19491	6.013759	-0.18159
H	-3.14849	6.201385	-0.44614
H	-4.79802	6.407084	-1.00852
C	-4.54818	6.768342	1.10679

H	-5.59401	6.599344	1.3831
H	-4.39136	7.843827	0.984022
H	-3.92245	6.426729	1.937308
C	-0.8735	3.699763	5.031938
H	-0.19069	4.496324	5.33849
H	-1.7186	3.710245	5.729023
C	-0.16115	2.34256	5.114985
H	-0.82821	1.538113	4.790499
H	0.168824	2.13379	6.137531
H	0.714855	2.335949	4.45981
C	-3.81437	3.096386	4.016075
H	-4.5207	2.545206	3.389331
H	-3.43002	2.36242	4.733108
C	-4.54839	4.226989	4.748464
H	-3.86263	4.794613	5.385864
H	-5.35399	3.833614	5.375908
H	-4.98449	4.926323	4.027605
O	1.154988	-5.24404	1.29881
O	1.411747	-4.79596	3.484809
N	-4.29041	-2.43322	0.712594
H	-3.96189	-1.63569	1.293865
N	-1.53015	-4.45669	1.458971
H	-1.26126	-4.73778	0.515101
C	-7.16358	-1.20384	-1.32859
H	-7.65548	-2.13886	-1.54899
C	-5.95353	-1.21105	-0.62684
C	-5.2695	-2.42183	-0.24873
C	-5.37187	-3.73345	-0.7601
C	-4.4362	-4.52621	-0.05858
C	-3.78583	-3.67996	0.844698
C	-2.72578	-3.92653	1.820837
C	-2.68666	-3.61172	3.17952
C	-1.39563	-3.95786	3.64547
C	-0.69949	-4.4702	2.549641
C	0.679429	-4.87701	2.366261
C	2.809391	-5.09486	3.350507
C	3.445472	-4.81947	4.696304
C	-6.25856	-4.25288	-1.85636

H	-5.71283	-5.03212	-2.40057
H	-6.46098	-3.46845	-2.59315
C	-7.56695	-4.85514	-1.32642
H	-8.13984	-4.12403	-0.74678
H	-8.1959	-5.22098	-2.14328
H	-7.34951	-5.69337	-0.6575
C	-4.2048	-6.00694	-0.18242
H	-3.15865	-6.19614	-0.44692
H	-4.80844	-6.39912	-1.00951
C	-4.55938	-6.76128	1.105735
H	-5.60498	-6.5907	1.381972
H	-4.40424	-7.83698	0.982715
H	-3.93321	-6.42086	1.936405
C	-0.88011	-3.69991	5.031911
H	-0.19829	-4.49748	5.338014
H	-1.72522	-3.70975	5.728996
C	-0.16606	-2.34364	5.115693
H	-0.83209	-1.53818	4.791591
H	0.164146	-2.1358	6.138353
H	0.709969	-2.3378	4.460544
C	-3.81988	-3.0913	4.016002
H	-4.52478	-2.53791	3.389588
H	-3.4342	-2.35894	4.733962
C	-4.55656	-4.22108	4.746986
H	-3.87221	-4.79075	5.384085
H	-5.36159	-3.82666	5.374504
H	-4.99383	-4.9188	4.025266
H	2.919918	-6.1418	3.04758
H	3.229252	-4.46824	2.555532
H	4.516901	-5.03412	4.655637
H	3.3108	-3.77133	4.975557
H	2.995825	-5.4454	5.471236
H	4.525369	5.025859	4.656149
H	3.004938	5.439054	5.471976
H	3.317297	3.764776	4.975345
H	3.23679	4.463178	2.555705
H	2.930064	6.136932	3.048727
H	-2.92994	-6.13731	-3.04827

H	-3.23685	-4.46349	-2.55558
H	-3.31775	-3.76555	-4.97529
H	-3.00498	-5.43983	-5.47169
H	-4.52546	-5.02691	-4.65582
H	-3.22926	4.470587	-2.55501
H	-2.91906	6.143842	-3.04758
H	-2.99545	5.446724	-5.47103
H	-3.31139	3.772998	-4.9748
H	-4.51673	5.036601	-4.65524
H	-4.55261	0.003659	0.269711
H	4.552639	-0.00382	-0.26998
Cl	-3.36763	0.00256	2.281475
Cl	3.367968	-0.0027	-2.28171
